# Experiences in education and interprofessional collaborative practice in multidisciplinary health residencies*

**DOI:** 10.1590/1980-220X-REEUSP-2025-0588en

**Published:** 2026-08-31

**Authors:** Lucas Cardoso dos Santos, Thiago da Silva Domingos, Eduardo Gabriel Cassola, Valéria Marli Leonello, Carolyne Reduzina Queirós, Marcelo Viana da Costa, Wilza Carla Spiri

**Affiliations:** 1 Universidade de São Paulo, Escola de Enfermagem, Departamento de Enfermagem em Saúde Coletiva, São Paulo, SP, Brazil.; 2 Universidade Estadual Paulista “Júlio de Mesquita Filho”, Faculdade de Medicina de Botucatu, Departamento de Enfermagem, Botucatu, SP, Brazil.; 3 Universidade de São Paulo, Escola de Enfermagem, Departamento de Orientação Profissional, São Paulo, SP, Brazil.; 4 Universidade Federal do Rio Grande do Norte, Escola Multicampi de Ciências Médicas, Caicó, RN, Brazil.

**Keywords:** Education, Interprofessional Education, Specialization, Interprofessional Relationships

## Abstract

**Objective:**

To understand the experiences of interprofessional collaborative education and practice among residents in multiprofessional health residency programs.

**Method:**

A descriptive, exploratory study with a qualitative approach. Eighteen residents enrolled in two residency programs at a Brazilian public university in the state of São Paulo participated. Data were collected through two synchronous remote meetings. The empirical data were analyzed using content analysis.

**Results:**

The themes highlighted opportunities for interprofessional education and practice, such as shared patient care, and the need to learn about and work with others. The difficulties faced by residents regarding healthcare work processes within the services were evident, as they operate under a multiprofessional framework.

**Final considerations:**

Experiences of interprofessional collaborative education and practice in multiprofessional residency programs occurred primarily in shared spaces for care and learning, fostering the development of collaborative competencies. However, organizational, pedagogical, and healthcare work process challenges still limit the consolidation of interprofessionality in training and practice.

## INTRODUCTION

Interprofessional Education (IPE) occurs when members of more than one health profession learn together in an integrated and interactive manner, with the explicit purpose of improving interprofessional collaboration and/or health care for patients, families, and communities^(1)^. It represents a powerful and innovative educational approach to training the health workforce, as it enables the development of collaborative competencies, such as interprofessional communication, user-, family-, and community-centered care, an understanding of professional roles and team dynamics, the resolution of interprofessional conflicts, and collaborative leadership^(1,2)^.

IPE facilitates Interprofessional Collaborative Practice (ICP), defined as collaboration in practice among members of two or more professions who work interactively, sharing objectives and recognizing each other’s roles and importance in complementary healthcare actions^(1)^. The literature shows that ICP strengthens healthcare services by improving the quality of care provided, greater user satisfaction, the commitment of the professionals involved in patient safety, the reduction of errors and duplication of procedures, the overcoming of stereotypes and lack of knowledge among professions regarding other professional fields, the sharing of knowledge for decision- making, and greater user involvement in care planning^(1,2,3,4,5)^.

Interprofessionality constitutes a theoretical, conceptual, and methodological framework that guides health education and practice aimed at providing comprehensive care centered on the needs of users, families, and communities. It is based on the understanding that interprofessional work encompasses different forms of interaction among professions—such as teamwork, collaboration, collaborative practice, and networking—and is not synonymous with teamwork. These forms differ in the degree of coordination of actions and interaction among professionals, as expressed through the sharing of values, goals, and identity^(6)^.

Within this spectrum, teamwork constitutes the most central dimension of interprofessional practice, as it involves greater interdependence, integration, and shared responsibility for care, and is essential for addressing the complexity of health needs. IPE, on the other hand, involves interactive and intentional learning among different professions, fostering the development of collaborative interprofessional practices and better health outcomes. Methodologically, it presupposes teaching strategies that promote interaction, dialogue, and shared knowledge construction, contributing to the development of collaborative competencies and to the improvement of care and health work processes^(1,2,3,4,5,6)^.

Globally, IPE emerged on the international scene in the 1960s and 1970s through joint training initiatives among different health professions, particularly in countries such as the United Kingdom and Canada, and was subsequently strengthened as a global agenda with the publication of the World Health Organization report in 2010. This milestone helped spread the concept of interactive interprofessional learning as a strategy for strengthening health systems, particularly primary care. Building on this movement, various international experiences began to highlight the potential of IPE to improve the quality of care, challenge fragmented training models, and promote collaborative practices, establishing itself as a relevant approach to overcoming the logic of professional silos in health education and practice^(1)^.

The Brazilian Unified Health System (SUS), guided by the principle of comprehensiveness, requires the formation of interprofessional teams capable of responding in a comprehensive and coordinated manner to the population’s health needs^(7)^. In this context, experiences with IPE at the national level, though still in their early stages, have been strengthened by the work of the Brazilian Network for Interprofessional Education and Work in Health (*Rede Brasileira de Educação e Trabalho Interprofissional em Saúde* – ReBETIS) and by policies driving changes in education, such as the Education Through Work for Health Program (*Programa de Educação pelo Trabalho para a Saúde* – PET-Saúde) and the Multiprofessional Health Residency Programs (*Programas de Residências Multiprofissionais em Saúde* – PRMS), which enhance integration among professions, foster collaborative practice, and contribute to the reorientation of health education in the country^(8)^.

The PRMS, regulated in Brazil in 2005, are examples of the *lato sensu* postgraduate education model, geared toward various health professions such as Biomedicine, Biological Sciences, Physical Education, Nursing, Pharmacy, Medical Physics, Physical Therapy, Speech-Language Pathology, Veterinary Medicine, Nutrition, Dentistry, Psychology, Social Work, Occupational Therapy, and Public Health, with the exception of medicine^(9,10)^.

PRMS programs aim to train professionals capable of working as part of a team, integrating knowledge and promoting comprehensive care aligned with health needs^(9,10)^. They contribute to the reformulation of the healthcare model by seeking more humanized standards that are better suited to meeting the population’s needs^(11,12)^. They offer opportunities for interprofessional work guided by the PIPE, which enables collaborative practice leading to better health outcomes^(12)^.

Multiprofessional Residency Programs represent a strategic area for the development of IPE and PCI by directly contributing to the training of professionals who are better prepared to face public health challenges with ethics and commitment, taking the health needs of users, families, and communities as their starting point^(11,12,13,14)^. In this sense, multiprofessional residencies constitute a public policy that drives the reorientation of health education, playing a central role in consolidating interprofessional practices within the SUS^(9)^. However, despite their recognized benefits, IPE and PCI, in the context of PRMS, are still scarcely explored in the literature^(12,13)^, which highlights the need to expand scientific research on interprofessionality in this setting.

Thus, incorporating IPE into the various curricula and training settings of health graduate programs represents a powerful approach to breaking down professional stereotypes and transforming the way health is practiced and conceptualized^(11,12,13,14)^, with PRMS offering a potential avenue for interprofessional education and practice. Thus, the research question for this study is: Are Multiprofessional Health Residency Programs effective settings for the development of interprofessional collaborative education and practice? To this end, the objective was to explore the experiences of interprofessional collaborative education and practice among residents in multiprofessional health residency programs.

## METHOD

### Study Type

This is a descriptive, exploratory, qualitative study^(15)^ that followed the recommendations of the *Consolidated Criteria for Reporting Qualitative Research* ^(16)^.

### Setting, Population, and Selection Criteria

The research setting was a public institution of higher education located in a municipality in the central-western region of the state of São Paulo, Brazil, with an estimated population of 149,718 inhabitants. It offers undergraduate programs in nursing and medicine, as well as graduate programs in both the *stricto sensu* and *lato sensu* categories, such as the Multidisciplinary Residency Programs in Family Health (Programas de Residência Multiprofissional em Saúde da Família – PRMSF) and Mental Health (Programas de Residência Multiprofissional em Saúde Mental – PRMSM), admission to which is determined through an annual selection process. These programs last two years and have a weekly course load of 60 hours and a total of 5,760 hours, divided between practical activities carried out in health care settings and theoretical and theoretical-practical activities conducted at the training institution.

These activities include specific training sessions by professional field and shared sessions among different professions, focused on case discussions, care planning, and reflection on the healthcare work process, which fosters experiences in interprofessional education and practice. This setting was chosen because these are well-established programs affiliated with a public institution recognized for its university-service-community integration.

The participants were residents enrolled in these programs, selected because they were directly involved in training processes that require interaction among different professions and teamwork; they were therefore considered key informants for understanding experiences related to interprofessional education and practice in the context of the multiprofessional residency.

The PRMSF comprises the professional categories of physical education, nursing, physical therapy, nutrition, dentistry, psychology, and social work, with four openings for the nursing program and one opening for each of the others, for a total of ten incoming residents per year. The PRMSM, on the other hand, includes nursing, psychology, social work, and occupational therapy, offering two openings per category per year, for a total of eight residents.

The residents’ practice settings vary across different points within the Health Care Networks (RAS), such as Primary Health Care Units, School Health Centers, and the Strategic Psychosocial Care, Deinstitutionalization, and Psychosocial Rehabilitation Services, among others. The programs involve coordinators, deputy coordinators, preceptors, tutors, and faculty members, none of whom receive *pró-labore* compensation for performing these roles. Certain services, because they host residents from more than one of the institution’s programs, have professionals with overlapping roles; that is, they sometimes act as preceptors and other times as tutors.

Eligible participants for the study were residents actively enrolled in the second year of the PRMSF and PRMSM programs who were interested in participating in the study. The exclusion criteria were: withdrawal from the residency program; extended sick leave; or vacation periods coinciding with the data collection phase. All residents in the aforementioned programs agreed to participate in the study, totaling 18 participants; therefore, there were no refusals.

### Data Collection

Data collection took place through a participatory workshop structured around two synchronous remote sessions, utilizing group dynamics and mediated collective discussion, aimed at exploring participants’ experiences and fostering reflections and shared meanings among them^(17)^. The workshop was planned in collaboration with the PRMSF and PRMSM coordinators to take place within the residents’ academic schedules, with the aim of ensuring greater participant engagement without interfering with the activities planned by the residency programs. Consideration was also given to holding the activity during periods when residents had shared curricular activities. Two synchronous remote meetings were held via a videoconferencing system (*Google Meet*); they were recorded, took place one week apart, and lasted approximately four hours each, in June 2021, amid the Coronavirus Disease 2019 (COVID-19) pandemic.

The first session began with the first author—a nurse with a master’s degree in public health—welcoming the participants. This was followed by an explanation of the study’s objectives, clarification of any questions, and the provision of an electronic form created in *Google Forms* for recording informed consent, in accordance with the guidelines of the National Research Ethics Commission^(18)^, as well as a self-administered questionnaire for sociodemographic characterization, including: gender, age, undergraduate degree (year of completion; name, city, and state of the educational institution; course format—in-person, blended, or distance learning) and the associated residency program.

The participants were then divided by residency program into two separate virtual rooms, with one facilitator per group: a nurse with a Ph.D. in nursing and a nurse with a master’s degree in nursing. The principal investigator moved between the two virtual rooms to address any questions and monitor the discussions. It is important to note that the facilitators and the principal investigator had prior experience in conducting qualitative research and had no prior close relationships with the study participants.

The objectives of the first session were: to assess the participants’ knowledge of IPE and PCI and to understand the teaching-learning-work process in both programs. Prompting questions were used to encourage residents to discuss in groups, followed by a plenary presentation to all workshop participants: (1) In what situations during residency activities do you have the opportunity to work in an interprofessional manner? (2) What characterizes these activities as interprofessional?; (3) In your opinion, what facilitates the practice of interprofessional work in the context of residency activities?; (4) In your opinion, what hinders the practice of interprofessional work in the context of residency activities?”

Each group collectively discussed and validated the points raised based on each guiding question. The participants’ comments complemented one another, with no apparent disagreements among the residents during the group discussions organized by residency program. Thus, the content produced by each group represented a consensus collectively reached by the participants of the same program. Subsequently, in a plenary session, a representative from each group presented a summary proposal, which was again discussed and collectively validated by the participants from both programs.

The second meeting began with the principal investigator, who first reviewed the research objectives, the summary proposal developed by the groups during the first meeting, and presented the work plan for the second meeting. Following this, an interactive presentation was given on the theoretical-conceptual and methodological framework of IPE and PCI by an expert in the field—a nurse with a Ph.D. in health sciences.

Subsequently, the residents were again divided into two groups by program, in separate virtual rooms, where they were asked to revisit the synthesis they had developed based on new guiding questions: (1) What were the most significant lessons learned from these experiences? Were these lessons stimulated or developed in different ways by the residents?; (2) What were the most explicit intentions behind these initiatives? How was this intent expressed?; (3) Which stages of the teaching-learning process facilitated the development of competencies for effective teamwork?; (4) At what point were the advantages and/or challenges discussed for the evaluation and planning of the teaching-learning process?

As a result, the responses provided during the first meeting were intentionally revisited through new questions, aiming to spark a group discussion based on the theoretical-conceptual and methodological framework of IPE that had been presented. After this, each group presented a new summary of the points discussed to the entire group.

At the end of the workshop, a *link* was provided to access the activity evaluation form. The first part contained a *Likert*-type scale with five response options (“strongly disagree,” “somewhat disagree,” “neutral,” “somewhat agree,” “strongly agree”) related to the workshop’s objectives, the workshop’s organization, the performance of the principal investigator, guest speaker, and facilitators, and the participants’ self-assessment. In the second part, participants were asked to complete the sentences beginning with the phrases: “I didn’t even expect it, but it happened…,” “I was a little disappointed with…,” “If I were to promote this workshop to a colleague, I would say that….”

### Data Analysis and Processing

The research data were organized according to the qualitative Content Analysis proposed by Graneheim et al.^(19)^. This analytical framework was operationalized through a reading of the text—comprising the transcripts of the two meetings and the notes taken by the facilitators—with the aim of extracting and compiling a new document that constituted the unit of analysis. Subsequently, units of meaning were identified—defined as words, phrases, or paragraphs—containing aspects related to one another through their content and context. These units were condensed, preserving their core meanings, and subsequently coded. Codes with similar themes were grouped, resulting in four themes (Chart 1). The interprofessionality framework^(1,2,3,4,5,6)^ was used as a reference for the discussion of the data.

**Chart 1:** Presentation of the framework, themes, units of meaning, and participant representation – Botucatu, SP, Brazil, 2021.

Framework	Topics	Units of meaning*	Representation of PRMS
Interprofissionalidade	Opportunities identified as promising for interprofessional education and practice	Shared care; Planning and implementation of joint activities; Discussions and case studies using the matrix support model; Service team meetings; Mentoring; Networking and intersectoral collaboration	PRMSM PRMSF
		Teaching strategy; Spontaneous and informal gatherings; Group activities involving all categories of the PRMSM	PRMSM
		Meetings between residents and the PRMSF coordination team; Development of: genogram, eco-map, and individualized treatment plan; Theoretical classes with other programs	PRMSF
	Learning outcomes developed through shared practice	New approaches to engaging clients; Identification of health needs; Development of interprofessional competencies; Dynamics of collaborative work; Results of the IPE and PCI	PRMSM
	Essential components for interprofessional education and practice	Availability for IPE and PCI; Understanding professional roles; Bonds among residents; Knowledge of IPE and PCI among tutors, preceptors, and program coordinators; Intentionality regarding IPE and PCI	PRMSM PRMSF
	Challenges to achieving interprofessional education and practice	Complexity of health needs; Service work processes; Resistance to change; Infrastructure; Staff turnover; Interference from municipal managers and program coordinators; Organization of program activities	PRMSM PRMSF

*The units of meaning presented here correspond to the group consensus reached and validated by the residents of each program.

### Ethical Considerations

This study was approved under decision number 3,961,371 on March 2, 2020, by the Research Ethics Committee of the sponsoring institution and followed all recommendations regarding research involving human subjects and any phase conducted in a virtual environment^(18,20)^. To use excerpts from the workshop participants’ statements, an alphanumeric code consisting of a digit followed by the program’s acronym (PRMSM, PRMSF) was employed, and any information that could identify them—such as professional category and the names of tutors, preceptors, and coordinators—was omitted.

## RESULTS

The participants’ ages ranged from 22 to 34 years, with the largest group being 25 years old; 12 participants identified as female and six as male. All had completed their undergraduate degrees through in-person programs at institutions in the state of São Paulo, with 16 having attended public institutions, mostly in 2019.

The workshop evaluation was considered positive, as the attributes related to the workshop’s objectives and organization, the performance of the principal investigator and facilitators, and the residents’ self-assessments were overwhelmingly rated as “strongly agree.”

The insights derived from the open-ended questions confirmed the initial reports, showing that participants reflected on clinical practice, residency training, and IPE and PCI, in addition to sharing their experiences. Despite frustrations with the current reality and the perception that interprofessionality is still far from being put into practice, they recognized the importance of the workshop for evaluating the teaching-learning-work process, sharing challenges, and reflecting on new ways of working as a team and providing health care.

The results of the analysis of the empirical data from the two meetings enabled the identification of units of meaning, which were subsequently condensed and grouped into four themes: Opportunities identified as conducive to interprofessional education and practice; Learning developed through shared practice; Components necessary for interprofessional education and practice; Challenges to achieving interprofessional education and practice.

### Opportunities Identified as Promising for Interprofessional Education and Practice

The first theme addresses the opportunities identified in residency activities that are considered promising for interprofessional practice. Among those featured in both programs are: shared patient care provided through consultations and home visits; the planning and execution of joint activities; discussions and case studies within the matrix support model; team meetings; mentoring; and networking and intersectoral collaboration.

*Shared patient care was a common topic of discussion in our group. Having the opportunity to provide care alongside another professional, to be there, to discuss that case, to discuss it more thoroughly afterward, and to be there as a source of support during the care encounter.* (3 PRMSM)

*Meetings are important because they allow us to review work processes [...] they facilitate more interprofessional collaboration during team meetings. This is important!*

*Understanding the work process, what these experiences entail, and what adds value to our training.* (15 PRMSF)

Discussions within the matrix support model were noted as powerful for interprofessional work, as they provide a space where collective thinking is developed and debated, rather than merely intending to refer the client to another professional.

PRMSM residents mentioned the use of the Problem-Based Learning teaching strategy and spontaneous, informal encounters during clinical practice. PRMSF residents, on the other hand, highlighted specific situations in addition to those already mentioned: meetings between residents and program coordinators; the development of genograms, eco-maps, and Individualized Treatment Plans (ITPs); the use of tools for family-centered care; and theoretical classes with residents from other residency programs.

PRMSM participants agreed that working in services as groups of residents alongside all professional categories within the program allows IPE to materialize more easily, unlike in the PRMSF, where the division of residents across practice settings prevents such a dynamic from occurring.

*Being close to the patients makes the work easier. We’re able to rotate through the practice settings as a mini-team. We’ll rotate through the services, but as a mini-team. And during on-call shifts, we’ll be together as one large mental health team. This facilitates our exchange and also fosters bonding and opportunities for discussion.* (3 PRMSM)

Being together at all times and in the same practice settings was cited as a way for residents to continuously participate in each other’s practice, understand how the group functions, plan and carry out planned activities, make decisions, solve problems more quickly, and recognize professional roles.

### Learning Developed Through Shared Practice

The second theme addresses residents’ learning in shared practice with professionals from different disciplines, highlighting the understanding of new ways of approaching clients, the identification of actual health needs, the recognition of colleagues’ competencies, and the dynamics of collaborative work, whether multi- or interprofessional.

*Shared patient care sessions allowed us to learn more about how other professionals work.* (6 PRMSM)

The residents noted that, over time, they were able to identify specific competencies among professionals and observed improvements in the quality of care and health outcomes. However, this collaborative care occurs more frequently among themselves than with service providers. Team discussions, despite conflicts, foster closer relationships, joint planning of actions, reflection on practice, conflict resolution, and a better understanding of how teams function.

*The joint activities we engage in are also helpful for resolving conflicts among us, including our differing opinions on the same case.* (1 PRMSM)

Both groups reported that the program’s activities equip residents for practice and were highlighted for enabling them to (re)think the work performed and develop collaborative skills, even if this was not explicitly named or stated by the participants.

### Essential Components for Interprofessional Education and Practice

The third theme encompasses the components and characteristics reported by the residents as essential elements for achieving interprofessional practice, such as seeking to understand the needs of others, the professional roles of peers, and learning from one another and as a team. They emphasized the need to understand that there is no absolute knowledge and that professionals must be able to fill gaps in their colleagues’ knowledge without judgment. There is, therefore, a need to be open to trusting what others are saying, acknowledging that there may be things one does not understand.

*Others need to understand that their knowledge and training will reach a limit, and at that limit, we need to work as a team so that we can expand the scope of care. On my own, I can only get so far—and beyond a certain point, how am I going to provide that care?* (3 PRMSM)

On the other hand, both groups highlighted the bond as a characteristic that facilitates interactions and allows insecurities and gaps in knowledge to be shared without fear of judgment by colleagues, thereby enabling collaborative learning. Personal openness to collaboration, the ability to listen, mutual respect among professionals, and trust in colleagues’ work and expertise were also characteristics highlighted by the residents.

*The other person needs to be open so that we can ask questions, talk, and discuss the issues that arise in practice. Openness is a very important factor—especially the willingness to listen.* (3 PRMSM)

Participants in both programs highlighted as distinguishing attributes the fact that preceptors, tutors, coordinators, and service managers have knowledge of PIPE and PCI. The lack of this knowledge is seen as a reason why some settings are perceived by residents as poor practice environments.

*When there is a deliberate intent and interest in making practices interprofessional, things in the residency programs unfold differently, but we do not see explicit planning of activities to be interprofessional. (9 PRMSF)*

*There is a tendency to be overwhelmed by work demands, which hinders intentionality. Intentionality is subordinated to demand.* (3 PRMSM)

They highlighted the need for a stated intent in the planning of interprofessional activities by those involved in the residency program; however, in practice, this logic is reversed, with the focus ultimately resting on service delivery demands rather than on the development of collaborative competencies.

### Challenges to Achieving Interprofessional Education and Practice

The fourth theme addresses the difficulties faced by residents in interprofessional practice, highlighting the complexity of users’ health needs, lack of time for discussions, the dominance of certain professional groups, resistance to change, the predominance of a multiprofessional approach, physical space limitations, high staff turnover, and interference by managers and coordinators in planned activities.

*Service management ends up placing greater demands on one professional category, and some professions end up being overlooked in discussion processes and shared care—and this makes it much harder to integrate new professionals into care. [...] It ends up rendering some professions invisible.* (3 PRMSM)

The high volume of patient care sessions always forces professionals into a pace that reflects other operational rationales and does not align with interprofessionality, thereby undermining the quality of healthcare work. There is also pressure on residents to meet service demands due to the shortage of professionals. This is sometimes accompanied by the perception that shared care sessions are less productive than individual ones.

*The high demand for services ends up pushing us toward a single-profession practice. And we have to manage this somehow, so interprofessional collaboration gets sidelined.* (7 PRMSM)

*There is a shortage of professionals in various fields of our practice. We are unable to complete our training because we are responsible for meeting the service demand. The lack of guaranteed democratic spaces makes it very difficult for us to engage in interprofessional work because we often end up handling the workload that would normally fall to a specific professional or the service itself.* (14 PRMSF)

Difficulties related to residency programs are also highlighted in the following comments: the lack of planned activities with other residency programs and the 60-hour weekly workload. As for those pertaining to the theoretical framework, the following were cited: the lack of understanding among preceptors, tutors, PRMS coordinators, and service managers regarding the planning of actions within an interprofessional framework; and the lack of understanding of the roles of the different professions and of the residents themselves.

This weakness lies not only in the difficulty of understanding the resident’s role within the service but also in the lack of familiarity with the professional core of certain professional categories, which further undermines the training process focused on interprofessionality and the integration of some residents into practice settings. Added to this issue is the lack of knowledge on the part of faculty, tutors, and preceptors regarding the implementation of IPE.

*A lack of knowledge about what the different areas of expertise entail hinders the organization of the work. [...] There are stigmas and entrenched beliefs about what certain professionals do.* (1 PRMSM)

*The lack of knowledge among preceptors, coordinators, and even the Coordination Office regarding interprofessional practice and its importance means that we do not have planned interprofessional activities in the residency program.*(12 PRMSF)

Other challenges were identified, such as the lack of compensation for tutors and preceptors, single-profession training in undergraduate health programs, the involvement of service professionals in residency program activities, and a weak evaluation process.

## DISCUSSION

By understanding the experiences of residents in the PRMS regarding IPE and PCI, it was observed that, although there are initiatives that promote training and collaborative work, structural, organizational, and pedagogical challenges still persist, limiting the consolidation of interprofessionality. The findings showed that interprofessionality is shaped in the day- to-day operations of health services, through the relationships and practices developed in training settings, and is influenced by work organization, interactions among individuals, and the institutional conditions present in health services. Thus, the collective spaces identified by the residents foster learning built on the shared experience of working in healthcare, allowing interprofessional collaboration to emerge from the ongoing interaction among different individuals, bodies of knowledge, and professional practices.

Residency programs represent prime settings for collaborative experiences among different health professions, such as in shared patient care, team meetings, mentoring, and network activities. As a training pillar, IPE enhances the development of both individual and collective competencies. However, its effective implementation still faces gaps in conceptual understanding, requiring continuous institutional support, adequate infrastructure, mastery of active methodologies, and structural and cultural changes in the field of health education to promote interaction among students from different programs, with the aim of breaking away from the paradigm of a predominantly single-professional and disciplinary curriculum^(21,22)^.

IPE depends on a stated pedagogical intent, structured strategies, and training for tutors and preceptors in order to be effectively implemented^(13)^; therefore, there is a growing need for frequent continuing education processes aligned with the guidelines and foundations of programs that contribute to health education. Despite the identified opportunities, participants highlighted the absence of explicit pedagogical planning focused on interprofessionality due to the lack of preparation among participants (preceptors, tutors, supervisors, and faculty), which undermines interprofessional initiatives.

Thus, the planning of teaching activities and the evaluation of the teaching-learning process are crucial and relevant components for the adoption of IPE. To this end, teaching plans must specify the number of hours allocated for scheduled meetings between tutors, preceptors, and residents, as these ensure the intentionality of the activities and recognize the importance of systematic and continuous assessment of learning and the acquisition of collaborative competencies, which include the student’s ability to work together with other professionals, patients, families, and communities^(23)^.

In this regard, limited interaction among tutors, preceptors, and residents may also hinder residents’ understanding of interprofessionality, as meetings among them could be guided by the IPE and ICP, approaches that remain largely underutilized in the programs analyzed. Thus, the collaborative experiences developed during the residency are directly related to the quality of interactions that occur in the day-to-day practice of healthcare and to the possibilities for the shared construction of care among the different stakeholders involved.

Learning in small groups with balanced representation of all professional categories involved presents itself as another strategy to facilitate interprofessional learning^(23)^, a feature observed only in the PRMSM program due to its equal number of slots per professional category. This finding indicates that the configuration of practice settings has a direct impact on the realization of interprofessionality.

Interprofessional practices enabled residents to recognize their colleagues’ expertise, critically reflect on care, and strengthen teamwork, thereby promoting an integrated education centered on the user’s needs. Such learning goes beyond the technical-operational dimension, involving relational and communicational processes built upon shared experiences of care and healthcare work. Thus, IPE and ICP emerge as promising approaches for enhancing comprehensive care by developing common, specific, and collaborative competencies, complementing—rather than replacing—multiprofessional, multidisciplinary, interdisciplinary, or even single-profession models^(24)^.

The PRMS represent an opportunity to break down professional stereotypes, as evidenced in the meetings, highlighting the importance of clear roles for effective collaborative work and the reconstruction of the specific knowledge of each profession. Recognition of the interdependence among these fields reinforces the importance of interprofessional communication as an essential competency for overcoming barriers, strengthening relationships, and improving care through shared decision-making^(25)^.

A lack of understanding regarding the role of certain professions emerged in the participants’ discourse. Some professions, being historically older, are better understood—albeit in a stereotypical manner—by various sectors of society, such as nursing and medicine. Others, such as occupational therapy, for example, are met with confusion among other professional groups and service users regarding their professional role^(26)^.

Some professional categories, encompassed by the programs analyzed here, are identified in the literature as those that face the greatest difficulty in integrating into service settings, such as speech-language pathology, nutrition, psychology, and occupational therapy. This situation is exacerbated by the expectations placed on these professionals, particularly those who work individually in specialized services—a practice that contrasts with the interprofessional approach—requiring a greater understanding of roles and alignment of objectives for the effective integration of these disciplines into health care practices^(27)^.

There are differing views on whether certain professional fields are more open and receptive to IPE. Tools such as the *Readiness for Interprofessional Learning Scale* (RIPLS) measure students’ readiness for shared learning with different professional groups by assessing three factors: teamwork and collaboration, professional identity, and patient-centered care^(28)^. However, there are discrepancies in the literature regarding the scales used in studies, which undermines the comparability of findings that analyze students and professionals longitudinally during and after an intervention^(23,29)^.

In a study aimed at understanding the experiences of graduates from a Family Medicine Residency Program (PRMSF), it was found that the residency program serves as a setting for learning from others and understanding their roles^(14,30)^. In contrast, a study analyzing the integration of PRMS programs into the structure of RAS found that these programs facilitate the implementation of networking, highlighting their importance for professional training^(30)^.

The presence of residents in the daily practice settings results in an increase in the workforce of these services and in the team’s capacity to address users’ health problems, while simultaneously creating confusion regarding their roles in training settings, as they are often viewed as a means to address the shortage of healthcare personnel—a deviation from the premise of PRMS^(30)^.

Added to this issue are other factors that impact teaching and learning in PRMS, including the discrepancy between the pedagogical approach and its practical application, a disconnect between the curriculum and field activities, interpersonal conflicts, stereotypes, a lack of recognition of roles, resistance to IPE, traditional training models, insufficient training of mentors, limited supervision, ineffective communication, and an emphasis on individual performance and competitiveness^(11,30)^. These factors demonstrate that the consolidation of interprofessionality depends not only on the individual willingness of professionals and residents but also on the organizational, pedagogical, and institutional conditions that shape work processes and health education. To change this reality, a joint effort is needed from training institutions, health services, and public health and education policies that value IPE as the foundation of health education.

The limitations of this study stem from the fact that the research was conducted at only one institution offering PRMS programs, as well as the non-participation of preceptors, tutors, coordinators, service users, and service professionals, which precludes an analysis of the context based on the experiences of all individuals involved in the practice and training settings of the residency programs. Added to this is the fact that data collection took place through remote, synchronous meetings, which may have influenced the interaction between researchers and participants, as well as the perception of nonverbal elements of communication.

The findings of this study highlight significant advances for nursing and the health field by reaffirming PRMS as strategic settings for the development of education and collaborative interprofessional practice. By highlighting learning related to interprofessional communication, role recognition, and shared decision-making, the study contributes to strengthening essential competencies for comprehensive care within the SUS. For the healthcare field, the findings broaden understanding of the pedagogical, organizational, and institutional factors that either promote or limit interprofessionality in in-service training settings, providing insights for improving educational programs, continuing education policies, and healthcare workforce management models.

Additionally, studies analyzing training processes and interprofessional dynamics in residency programs are of a structural nature rather than merely circumstantial, as they address organizational, pedagogical, and relational dimensions that tend to persist over time, even in the face of changing circumstances. Thus, the findings of this research remain current and relevant, contributing to the advancement of knowledge in the field, supporting the improvement of residency programs, and aiding in the formulation and refinement of public policies aimed at health education.

## FINAL CONSIDERATIONS

This study provided insight into residents’ experiences with interprofessional education and collaborative practice in multiprofessional health residency programs, highlighting training opportunities, learning outcomes, necessary components, and challenges for achieving interprofessionality in on-the-job training settings. The findings showed that these experiences primarily take place in everyday settings of interaction between residents and service teams—such as shared patient care, case discussions, and team meetings—which foster the recognition of professional roles and the development of collaborative skills.

However, the results also revealed that the consolidation of interprofessionality is still hindered by structural, organizational, and pedagogical aspects of health services and residency programs, such as the predominance of a multiprofessional approach to training and healthcare work, the high demand for patient care within these services, and the lack of intentional planning for interprofessional activities. Thus, it is imperative that IPE be implemented across various educational processes, breaking away from traditional paradigms of teaching and healthcare practice, with the aim of improving healthcare and collaboration among professionals.

Finally, the importance of research that considers different contexts, approaches, and populations is highlighted, especially longitudinal, interventionist, and mixed-methods studies, which are still scarce in the field of interprofessional education and practice. It is believed that new research could inform changes in education and the formulation of new public health and education policies.

## Data Availability

The entire dataset supporting the results of this study was published in the article itself.
