# Supplementary material for: Experiences in education and interprofessional collaborative practice in multidisciplinary health residencies
Source: Rev Esc Enferm USP. 2026 Aug 31;60:e20250588. doi: 10.1590/1980-220X-REEUSP-2025-0588en (PMC13537110; doi:10.1590/1980-220X-REEUSP-2025-0588en)
Supplement: Translation

# Translation: Vivências da educação e prática colaborativa interprofissional em residências multiprofissionais em saúde*

**DOI:** 10.1590/1980-220X-REEUSP-2025-0588pt

**Authors:** Lucas Cardoso dos Santos, Thiago da Silva Domingos, Eduardo Gabriel Cassola, Valéria Marli Leonello, Carolyne Reduzina Queirós, Marcelo Viana da Costa, Wilza Carla Spiri

**Affiliations:** 1 Universidade de São Paulo, Escola de Enfermagem, Departamento de Enfermagem em Saúde Coletiva, São Paulo, SP, Brasil.; 2 Universidade Estadual Paulista “Júlio de Mesquita Filho”, Faculdade de Medicina de Botucatu, Departamento de Enfermagem, Botucatu, SP, Brasil.; 3 Universidade de São Paulo, Escola de Enfermagem, Departamento de Orientação Profissional, São Paulo, SP, Brasil.; 4 Universidade Federal do Rio Grande do Norte, Escola Multicampi de Ciências Médicas, Caicó, RN, Brasil.

**Keywords:** Educação, Educação Interprofissional, Especialização, Relações Interprofissionais

## Abstract

**Objetivo:**

Conhecer as vivências de educação e prática colaborativa interprofissional de residentes de programas de residência multiprofissional em saúde.

**Método:**

Estudo descritivo, exploratório, de abordagem qualitativa. Participaram 18 residentes vinculados a dois programas de residência de uma universidade pública brasileira do estado de São Paulo. A coleta de dados ocorreu por meio de dois encontros remotos síncronos. A análise do material empírico foi feita a partir da Análise de Conteúdo.

**Resultados:**

Os temas elucidaram as oportunidades para a educação e prática interprofissional, como atendimentos compartilhados, e a necessidade de querer conhecer sobre e atuar com o outro. Evidenciaram-se as dificuldades enfrentadas pelos residentes voltadas aos processos de trabalho em saúde dos serviços, por operarem sob uma lógica multiprofissional.

**Considerações finais:**

As vivências de educação e prática colaborativa interprofissional nas residências multiprofissionais ocorreram principalmente em espaços compartilhados de cuidado e aprendizagem, favorecendo o desenvolvimento de competências colaborativas. Contudo, desafios organizacionais, pedagógicos e dos processos de trabalho em saúde ainda limitam a consolidação da interprofissionalidade na formação e trabalho.

## INTRODUÇÃO

A Educação Interprofissional (EIP) ocorre quando os membros de mais de uma profissão da saúde aprendem em conjunto, de forma integrada e interativa, com o propósito explícito de melhorar a colaboração interprofissional e/ou o cuidado em saúde de usuários, famílias e comunidades^(1)^. Apresenta-se como uma abordagem educacional inovadora potente para a formação da força de trabalho em saúde por permitir o desenvolvimento de competências colaborativas, como a comunicação interprofissional, o cuidado centrado no usuário, na família e na comunidade, a compreensão dos papéis profissionais e da dinâmica de organização da equipe, a resolução de conflitos interprofissionais e a liderança colaborativa^(1,2)^.

A EIP proporciona uma Prática Colaborativa Inter­profissional (PCI), compreendida como a colaboração na prática entre membros de duas ou mais profissões que atuam de forma interativa, compartilhando objetivos e reconhecendo o papel e a importância do outro na complementaridade das ações em saúde^(1)^. A literatura evidencia que a PCI fortalece os serviços de saúde a partir da melhora na qualidade do cuidado em saúde ofertado, da maior satisfação do usuário, do comprometimento dos profissionais envolvidos com a segurança do paciente, da redução de erros e da duplicidade de procedimentos, da superação de estereótipos e do desconhecimento entre as profissões sobre as demais áreas profissionais, do compartilhamento de saberes para a tomada de decisões e do maior envolvimento dos usuários no planejamento do cuidado^(1,2,3,4,5)^.

A interprofissionalidade constitui um referencial teórico-conceitual e metodológico orientador da formação e do trabalho em saúde voltados à produção do cuidado integral e centrado nas necessidades dos usuários, famílias e comunidades. Fundamenta-se na compreensão de que o trabalho interprofissional abrange diferentes modalidades de interação entre profissões — como trabalho em equipe, colaboração, prática colaborativa e trabalho em rede — não sendo sinônimo de trabalho em equipe. Essas modalidades se diferenciam pelo grau de articulação das ações e de interação entre os agentes, expressos no compartilhamento de valores, objetivos e identidade^(6)^.

Nesse espectro, o trabalho em equipe constitui a dimensão mais nuclear do trabalho interprofissional, por envolver maior interdependência, integração e responsabilização conjunta pelo cuidado, sendo fundamental para responder à complexidade das necessidades de saúde. Já a EIP envolve a aprendizagem interativa e intencional entre diferentes profissões, favorecendo o desenvolvimento de práticas colaborativas interprofissionais e melhores resultados em saúde. Metodologicamente, pressupõe estratégias de ensino que promovam interação, diálogo e construção compartilhada, contribuindo para o desenvolvimento de competências colaborativas e para a qualificação do cuidado e dos processos de trabalho em saúde^(1,2,3,4,5,6)^.

Mundialmente, a EIP emerge no cenário internacional, nas décadas de 1960 e 1970, a partir de iniciativas de formação conjunta entre diferentes profissões da saúde, especialmente em países como Reino Unido e Canadá, sendo posteriormente fortalecida como agenda global com a publicação do relatório da Organização Mundial da Saúde em 2010. Esse marco contribuiu para difundir a proposta de aprendizagem interativa entre profissões como estratégia para o fortalecimento dos sistemas de saúde, em especial da Atenção Primária. A partir desse movimento, diferentes experiências internacionais passaram a evidenciar o potencial da EIP para qualificar a assistência, tensionar modelos formativos fragmentados e promover práticas colaborativas, consolidando-se como uma abordagem relevante para superar a lógica de silos profissionais na formação e no trabalho em saúde^(1)^.

No Brasil, o Sistema Único de Saúde (SUS), orientado pelo princípio da integralidade, requer a composição de equipes interprofissionais capazes de responder de forma abrangente e coordenada às necessidades de saúde da população^(7)^. Nesse contexto, as experiências de EIP no cenário nacional, embora ainda incipientes, foram fortalecidas pela atuação da Rede Brasileira de Educação e Trabalho Interprofissional em Saúde (ReBETIS) e por políticas indutoras de mudanças na formação, como o Programa de Educação pelo Trabalho para a Saúde (PET-Saúde) e os Programas de Residências Multiprofissionais em Saúde (PRMS), que ampliam a integração entre profissões, qualificam a prática colaborativa e contribuem para a reorientação da formação em saúde no país^(8)^.

Os PRMS, regulamentados em 2005 no Brasil, são exemplos da modalidade de ensino de pós-graduação *lato sensu*, voltada a diversas profissões da saúde – Biomedicina, Ciências Biológicas, Educação Física, Enfermagem, Farmácia, Física Médica, Fisioterapia, Fonoaudiologia, Medicina Veterinária, Nutrição, Odontologia, Psicologia, Serviço Social, Terapia Ocupacional e Saúde Coletiva -, com exceção da medicina^(9,10)^.

Os PRMS têm como objetivo formar profissionais capazes de trabalhar em equipe, integrando saberes e promovendo um cuidado integral alinhado às necessidades de saúde^(9,10)^. Contribuem para a reformulação do modelo de atenção à saúde ao buscar por padrões mais humanizados e adequados ao atendimento das necessidades da população^(11,12)^. Oferecem oportunidades de trabalho interprofissional, orientado pela EIP, o que possibilita uma prática colaborativa com melhores resultados em saúde^(12)^.

Os PRMS representam um campo estratégico para o desenvolvimento da EIP e da PCI ao contribuírem diretamente na formação de profissionais mais preparados para enfrentar os desafios da saúde pública com ética e compromisso, tomando como ponto de partida as necessidades de saúde dos usuários, famílias e comunidades^(11,12,13,14)^. Nesse sentido, as residências multiprofissionais constituem-se como uma política pública indutora da reorientação da formação em saúde, assumindo papel central na consolidação de práticas interprofissionais no SUS^(9)^. Contudo, apesar dos reconhecidos benefícios, a EIP e a PCI, no contexto dos PRMS, ainda são escassamente exploradas na literatura^(12,13)^, o que evidencia a necessidade de ampliar a produção científica sobre a interprofissionalidade nesse cenário.

Dessa forma, incorporar a EIP nos diferentes currículos e cenários de formação nas pós-graduações em saúde configura-se como uma abordagem potente para a quebra de estereótipos das profissões e no modo de fazer e pensar saúde^(11,12,13,14)^, em que os PRMS se apresentam como uma possibilidade para o ensino e prática interprofissional. Assim, tem-se como pergunta para essa pesquisa: os Programas de Residências Multiprofissionais em Saúde são espaços potentes para o desenvolvimento da educação e prática colaborativa interprofissional? Para tanto, objetivou-se conhecer as vivências de educação e prática colaborativa interprofissional de residentes de programas de residência multiprofissional em saúde.

## MÉTODO

### Tipo do Estudo

Trata-se de uma pesquisa descritiva, exploratória, de natureza qualitativa^(15)^ que considerou as recomendações do *Consolidated Criteria for Reporting Qualitative Research* ^(16)^.

### Local, População e Critérios de Seleção

O cenário da pesquisa foi uma Instituição de Ensino Superior pública, localizada em um município do centro-oeste do Estado de São Paulo, Brasil, com população estimada de 149.718 habitantes. Oferta cursos de graduação em enfermagem e medicina e pós-graduação nas modalidades *stricto sensu* e *lato sensu*, como os Programas de Residência Multiprofissional em Saúde da Família (PRMSF) e Saúde Mental (PRMSM), cujo ingresso se dá por meio de processo seletivo anual. Esses programas têm duração de dois anos e possuem carga horária semanal de 60 horas e total de 5.760 horas, distribuídas entre atividades práticas desenvolvidas nos serviços de saúde e atividades teóricas e teórico-práticas realizadas na instituição formadora.

Tais atividades incluem momentos formativos específicos por área profissional e espaços compartilhados entre diferentes profissões, voltados à discussão de casos, planejamento do cuidado e reflexão sobre o processo de trabalho em saúde, o que favorece experiências de educação e prática interprofissional. A escolha desse cenário justificou-se por se tratar de programas consolidados, vinculados a uma instituição pública reconhecida pela integração universidade-serviço-comunidade.

Os participantes foram residentes vinculados a esses programas, selecionados por estarem diretamente inseridos em processos formativos que demandam interação entre diferentes profissões e trabalho em equipe, sendo, portanto, considerados informantes-chave para a compreensão das vivências relacionadas à educação e prática interprofissional no contexto da residência multiprofissional.

O PRMSF é composto pelas categorias profissionais educação física, enfermagem, fisioterapia, nutrição, odontologia, psicologia e serviço social, com quatro vagas para o curso de enfermagem e uma vaga para as demais, perfazendo um total de dez residentes ingressantes por ano. Já o PRMSM inclui a enfermagem, psicologia, serviço social e terapia ocupacional com oferecimento de duas vagas por categoria por ano, totalizando oito residentes.

Os cenários de atuação dos residentes variam entre os diferentes pontos das Redes de Atenção à Saúde (RAS), como Unidades Básicas de Saúde, Centros de Saúde Escola e os Serviços de Atenção Psicossocial Estratégica, Desinstitucionalização e Reabilitação Psicossocial, dentre outros. Apresentam coordenadores, vice coordenadores, preceptores, tutores e docentes envolvidos nos programas, sem o recebimento de *pró-labore* para o exercício dessas funções. Determinados serviços por receberem residentes de mais de um programa da instituição possuem profissionais com funções sobrepostas, ou seja, ora atuam como preceptores, ora como tutores.

Os participantes elegíveis para a pesquisa foram os residentes com matrículas ativas no segundo ano dos PRMSF e PRMSM com interesse em colaborar com a pesquisa. Já os critérios de exclusão foram: desligamento da residência; licença saúde prolongada ou período de férias coincidentes com a etapa de coleta de dados. Todos os residentes dos referidos programas aceitaram participar da pesquisa, o que totalizou 18 participantes, não ocorrendo, portanto, recusas.

### Coleta de Dados

A coleta de dados ocorreu por meio de uma oficina participativa estruturada em dois encontros remotos síncronos, com utilização de dinâmicas grupais e discussão coletiva mediada, voltada à problematização das vivências e à construção de reflexões e significados entre os participantes^(17)^. Foi planejada junto às coordenações dos PRMSF e PRMSM para que ocorresse dentro da carga horária das atividades acadêmicas dos residentes com vistas a garantir maior adesão dos participantes, sem prejuízo às atividades planejadas pelos programas de residência. Ponderou-se, também, a realização da atividade em períodos em que os residentes possuíam atividades curriculares compartilhadas. Foram realizados dois encontros remotos síncronos agendados por meio de um sistema de videoconferência (*Google Meet*), gravados, com intervalo entre eles de uma semana e duração aproximada de quatro horas cada, no mês de junho de 2021, em meio à pandemia do *Coronavirus Disease* 2019 (COVID-19).

O primeiro encontro iniciou-se com a recepção dos participantes pelo primeiro autor, enfermeiro e mestre em saúde coletiva. Após, houve a explicação dos objetivos da pesquisa, o esclarecimento de possíveis dúvidas e a oferta de um formulário eletrônico, elaborado no *Google Forms,* para o registro do consentimento livre e esclarecido, conforme orientações da Comissão Nacional de Ética em Pesquisa^(18)^, bem como um questionário autoaplicável para caracterização sociodemográfica, contendo: gênero, idade, graduação (ano de conclusão; nome, cidade e estado da instituição formadora; modalidade do curso – presencial, semipresencial ou ensino à distância) e o programa de residência vinculado.

Seguiu-se com a divisão dos participantes por programa de residência em duas salas virtuais distintas com a condução de um mediador por grupo: um enfermeiro e doutor em enfermagem e uma enfermeira e mestre em enfermagem. O pesquisador principal percorreu os dois ambientes para sanar possíveis dúvidas e acompanhar as discussões. Importa ressaltar que mediadores e pesquisador apresentam experiência prévia com o desenvolvimento de pesquisa qualitativa e não possuíam relações prévias e próximas com os participantes da pesquisa.

Os objetivos do primeiro encontro foram: levantar os conhecimentos dos participantes acerca da EIP e PCI e conhecer o processo ensino-aprendizagem-trabalho em ambos os programas. Utilizou-se de perguntas disparadoras para que os residentes debatessem em grupo com posterior apresentação em plenária a todos os envolvidos na oficina: (1) Em que situações nas atividades da residência você tem a oportunidade de atuar de forma interprofissional? (2) O que caracteriza essas atividades como interprofissionais?; (3) Na sua opinião, o que facilita a prática do trabalho interprofissional no contexto das atividades da residência?; (4) Na sua opinião, o que dificulta a prática do trabalho interprofissional no contexto das atividades da residência?”.

Cada grupo debateu e validou coletivamente os pontos levantados a partir de cada norteadora. As falas dos participantes foram complementares entre si, não havendo divergências manifestas entre os residentes durante as discussões grupais por programa de residência. Dessa forma, os conteúdos produzidos por cada grupo representaram consensos construídos coletivamente pelos participantes de um mesmo programa. Posteriormente, em plenária, um representante de cada grupo apresentou uma proposta-síntese, a qual foi novamente discutida e validada coletivamente pelos participantes dos dois programas.

O segundo encontro ocorreu com o pesquisador principal, inicialmente, retomando os objetivos da pesquisa, a proposta-síntese elaborada pelos grupos no primeiro encontro e apresentando a proposta de trabalho para o segundo encontro. Após esse momento foi realizada uma apresentação dialogada acerca do referencial teórico-conceitual e metodológico da EIP e PCI por um especialista na temática, enfermeiro e doutor em ciências da saúde.

Posteriormente, os residentes foram novamente divididos em dois grupos por programa, em salas virtuais distintas, em que deveriam revisitar a síntese construída a partir de novas perguntas norteadoras: (1) Quais as aprendizagens mais marcantes dessas vivências? Elas foram estimuladas ou desenvolvidas de diferentes formas pelos residentes?; (2) Qual(is) a(s) intencionalidades mais explícitas dessas iniciativas? Como essa intencionalidade se expressou?; (3) Quais etapas do processo ensino-aprendizagem facilitaram o desenvolvimento de competências para o efetivo trabalho em equipe?; (4) Em que momento as facilidades e/ou dificuldades foram discutidas para avaliação e planejamento do processo ensino-aprendizagem?

Dessa forma, intencionalmente, as respostas dadas no primeiro encontro foram revisitadas a partir de novas questões, buscando despertar uma discussão no grupo a partir do referencial teórico-conceitual e metodológico da EIP apresentados. Cada grupo após esse momento, apresentou em plenária uma nova síntese dos pontos debatidos.

Ao final da oficina, foi disponibilizado um *link* para acesso ao formulário para avaliação da atividade. A primeira parte continha uma escala tipo *likert* com cinco critérios (discordo totalmente, discordo parcialmente, neutro, concordo parcialmente, concordo totalmente) relacionados aos objetivos da oficina, organização da oficina, atuação do pesquisador principal, convidado e mediadores e autoavaliação dos participantes. Na segunda parte os participantes deveriam dar continuidade às frases iniciadas pelas expressões: “Eu nem esperava e aconteceu...”, “Decepcionei-me um pouco com...”, “Se eu fosse fazer uma propaganda a um colega sobre essa oficina eu diria que...”.

### Análise e Tratamento dos Dados

Os dados da pesquisa foram organizados segundo a Análise de Conteúdo proposta por Graneheim et al.^(19)^ na vertente qualitativa. A operacionalização desse referencial de análise se deu a partir da leitura do texto, representado pela transcrição das gravações dos dois encontros e das anotações feitas pelos mediadores com a intencionalidade de extrair e reunir um novo documento que constituiu a unidade de análise. Posteriormente, foram identificadas unidades de significados, compreendidas como palavras, frases ou parágrafos, contendo aspectos relacionados entre si através de seu conteúdo e contexto. Essas unidades foram condensadas, preservando seus núcleos de sentido e, posteriormente codificadas. Os códigos com aproximação temática foram agrupados, originando quatro temas (Quadro 1). Para a discussão dos dados considerou-se o referencial da interprofissionalidade^(1,2,3,4,5,6)^.

**Quadro 1:** Apresentação do referencial, dos temas, das unidades de significado e representação dos participantes – Botucatu, SP, Brasil, 2021.

Referencial	Temas	Unidades de Significado*	Representação dos PRMS
Interprofissionalidade	Oportunidades identificadas como potentes para o ensino e prática interprofissional	Atendimentos compartilhados; Planejamento e a execução de atividades comuns; Discussões e estudos de caso no modelo de apoio matricial; Reuniões de equipe do serviço; Tutoria; Trabalho em rede e intersetorial	PRMSM PRMSF
		Estratégia de ensino; Encontros espontâneos e informais; Atuação em grupos com todas as categorias do PRMSM	PRMSM
		Reuniões entre residentes e coordenação do PRMSF; Elaboração: genograma, ecomapa, projeto terapêutico singular; Aulas teóricas com outros programas	PRMSF
	Aprendizagens desenvolvidas por meio da prática compartilhada	Novas formas de abordar o usuário; Identificação das necessidades de saúde; Desenvolvimento das competências interprofissionais; Dinâmica do trabalho colaborativo; Resultados da EIP e PCI	PRMSM
	Componentes necessários para o ensino e prática interprofissional	Disponibilidade para a EIP e PCI; Conhecer os papéis profissionais; Vínculo entre os residentes; Conhecimento sobre a EIP e PCI pelos tutores, preceptores e coordenação; Intencionalidade para a EIP e PCI	PRMSM PRMSF
	Desafios para a consecução do ensino e prática interprofissional	Complexidade das necessidades de saúde; Processos de trabalho dos serviços; Resistência a mudanças; Infraestrutura; Rotatividade de profissionais; Interferência de gestores municipais e coordenadores dos programas; Organização das atividades dos programas	PRMSM PRMSF

*As unidades de significado apresentadas correspondem aos consensos grupais construídos e validados pelos residentes de cada programa.

### Aspectos Éticos

A presente pesquisa foi aprovada sob o número do parecer 3.961.371 em 02/03/2020 pelo Comitê de Ética em Pesquisa da instituição proponente e seguiu todas as recomendações voltadas às pesquisas que envolvem seres humanos e qualquer etapa em ambiente virtual^(18,20)^. Para utilização de excertos das falas dos participantes na oficina foi utilizado código alfanumérico composto por um algarismo sucedido pela sigla do programa (PRMSM, PRMSF), sendo suprimida qualquer informação que pudesse identificá-los, como a categoria profissional e o nome de tutores, preceptores e coordenadores.

## RESULTADOS

A idade dos participantes variou entre 22 e 34 anos com maior representação daqueles com 25 anos; 12 pessoas se identificaram como sendo do gênero feminino e seis do gênero masculino. Todos concluíram a graduação na modalidade presencial em instituições do estado de São Paulo, sendo 16 em instituições públicas e em grande parte no ano de 2019.

A avaliação da oficina foi considerada positiva, visto que os atributos relacionados aos objetivos e a organização da oficina, a atuação do pesquisador principal e mediadores e a autoavaliação dos residentes foram analisados em sua grande maioria com o critério “concordo totalmente”.

As ponderações oriundas das questões abertas confirmaram os relatos iniciais, mostrando que os participantes refletiram sobre a prática, a formação na residência e a EIP e PCI, além de trocarem vivências. Apesar das frustrações com a realidade e a percepção de que a interprofissionalidade ainda está distante da prática, reconheceram a importância da oficina para avaliar o processo ensino-aprendizagem-trabalho, compartilhar dificuldades e refletir sobre novas formas de atuação em equipe e cuidado em saúde.

Os resultados da análise do material empírico proveniente dos dois encontros possibilitaram a identificação de unidades de significado, posteriormente condensadas e agrupadas em quatro temas: Oportunidades identificadas como potentes para a educação e prática interprofissional; Aprendizagens desenvolvidas por meio da prática compartilhada; Componentes necessários para a educação e prática interprofissional; Desafios para a consecução da educação e prática interprofissional.

### Oportunidades Identificadas Como Potentes Para a Educação e Prática Interprofissional

O primeiro tema aborda as oportunidades identificadas nas atividades da residência compreendidas como potentes para uma atuação interprofissional. Dentre aquelas que figuram nos dois programas, têm-se: os atendimentos compartilhados realizados por meio de consultas e visitas domiciliares, o planejamento e a execução de atividades comuns, as discussões e os estudos de caso no modelo de apoio matricial, reuniões de equipe, tutoria e o trabalho em rede e intersetorial.

*Foi uma fala comum no nosso grupo os atendimentos compartilhados. Ter a possibilidade de atender com o outro, de estar ali, de discutir aquela demanda, discutir com mais propriedade depois, de estar ali como apoio também, suporte no momento do atendimento.* (3 PRMSM)

*As reuniões são importantes por permitir revermos os processos de trabalho [...] é um facilitador para que se trabalhe mais interprofissional nas reuniões de equipe. É importante isso! Entender o processo de trabalho, o que são essas vivências e o que agrega valor para a nossa formação.* (15 PRMSF)

As discussões no modelo de apoio matricial foram lembradas como potentes para o trabalho interprofissional, por se apresentarem como espaço em que o pensamento conjunto é elaborado e debatido, e não apenas tem-se a intenção de encaminhar o usuário a outro profissional.

Os residentes do PRMSM mencionaram a utilização da estratégia de ensino Aprendizagem Baseada em Problemas e os encontros espontâneos e informais da prática do trabalho. Já os residentes do PRMSF trouxeram situações particulares além das já citadas: as reuniões entre residentes e coordenação do programa, a elaboração do genograma, ecomapa e Projeto Terapêutico Singular (PTS), a utilização de instrumentos para abordagem familiar e as aulas teóricas com residentes de outros programas de residência.

Os participantes do PRMSM convergiram ao mencionar que o fato de atuarem nos serviços em grupos de residentes com todas as categorias profissionais do programa permite que a EIP se materialize com maior facilidade, diferente do PRMSF em que a divisão dos residentes pelos cenários de prática ocorre de forma a não permitir característica similar.

*Estar próximos dos sujeitos facilita o trabalho. Conseguimos rodar os campos de prática enquanto uma mini-equipe. Nós vamos rodar os serviços, mas enquanto uma mini-equipe. E nos espaços de plantão nós vamos estar como um grande grupo da saúde mental. Isso facilita nossa troca, também, permite o vínculo e os espaços de discussão.* (3 PRMSM)

Estar sempre juntos e nos mesmos cenários de prática foi referido como forma do residente participar da prática do outro de forma contínua, entender o funcionamento do grupo, planejar e dar continuidade nas atividades planejadas, tomar decisões, resolver problemas de forma mais rápida e (re)conhecer os papéis profissionais.

### Aprendizagens Desenvolvidas por meio da Prática Compartilhada

O segundo tema aborda as aprendizagens dos residentes na prática compartilhada com profissionais de diferentes categorias, destacando a compreensão de novas formas de abordagem ao usuário, a identificação das necessidades reais de saúde, o reconhecimento das competências dos colegas e a dinâmica do trabalho colaborativo, seja multi ou interprofissional.

*Os atendimentos compartilhados permitiram aprender mais sobre o modo de fazer dos demais profissionais.* (6 PRMSM)

Os residentes destacaram que, com o tempo, conseguiram identificar competências específicas dos profissionais e perceberam melhorias na qualidade do cuidado e nos resultados em saúde. Porém, esse atendimento colaborativo acontece mais entre eles do que com os profissionais dos serviços. As discussões em equipe, apesar dos conflitos, promovem aproximação, construção conjunta de ações, reflexão da prática, resolução de conflitos e melhor compreensão do funcionamento das equipes.

*As atividades em conjunto que temos é também boa para resolvermos conflitos que existem entre nós, inclusive nas nossas opiniões diferentes sobre um mesmo caso.* (1 PRMSM)

Ambos os grupos relataram que as atividades dos programas instrumentalizam os residentes na e para a prática e foram destacadas por permitirem (re)pensar o trabalho executado e o desenvolvimento das competências colaborativas, mesmo que sem a nomeação explícita ou declarada pelos participantes.

### Componentes Necessários Para a Educação e Prática Interprofissional

O terceiro tema abarca os componentes e as características relatados pelos residentes como elementos inerentes para o alcance de uma prática interprofissional, como querer conhecer a necessidade do outro, os papéis profissionais dos pares e aprender com o outro e em equipe. Destacaram a necessidade do entendimento de que não há saberes absolutos e que o profissional deve estar apto a colaborar nas lacunas do conhecimento dos colegas sem julgamentos. Há a necessidade, portanto, de ceder para confiar no que o outro está afirmando, assumindo o lugar de não entendimento sobre algo.

*O outro tem que entender que o conhecimento dele, a formação dele, vai chegar em um limite e nesse limite a gente precisa trabalhar enquanto equipe para que possamos ampliar esse cuidado. Sozinho eu posso chegar até num momento e a partir de um ponto como é que eu vou propiciar esse cuidado?* (3 PRMSM)

Por outro lado, o vínculo foi lembrado pelos dois grupos como característica que facilita as interações e oportuniza que as inseguranças e os desconhecimentos sejam postos sem receio do julgamento pelo colega, possibilitando assim uma aprendizagem colaborativa. A disponibilidade pessoal para a colaboração, o saber ouvir, o respeito entre os profissionais, a confiança no trabalho e no saber do colega também foram características acrescentadas pelos residentes.

*O outro precisa estar disponível para que a gente possa questionar, conversar, discutir as questões que permeiam a prática. A disponibilidade é um fator muito importante. Principalmente o do ouvir.* (3 PRMSM)

Os participantes dos dois programas destacaram como atributos diferenciais os preceptores, tutores, coordenadores e gestores dos serviços terem conhecimento acerca da EIP e PCI. A falta deste é tida como justificativa para que alguns cenários sejam percebidos como campos de prática ruins pelos residentes.

*Quando tem intencionalidade e interesse de fazer das práticas algo interprofissional as coisas na Residência acontecem diferente, mas não vemos um planejamento das atividades para serem interprofissionais de forma explícita. (9 PRMSF)*

*Há uma captura pelas demandas do trabalho que dificulta a intencionalidade. A intencionalidade é subordinada pela demanda.* (3 PRMSM)

Evidenciaram a necessidade de haver uma intencionalidade declarada no planejamento de atividades interprofissionais por parte dos sujeitos envolvidos na residência, mas que na prática há uma inversão nessa lógica que acaba por residir nas demandas por atendimento dos serviços e não no desenvolvimento das competências colaborativas.

### Desafios Para a Consecução da Educação e Prática Interprofissional

O quarto tema aborda as dificuldades enfrentadas pelos residentes na prática interprofissional, destacando a complexidade das necessidades de saúde dos usuários, falta de tempo para discussões, hegemonia de algumas categorias profissionais, resistência a mudanças, predominância da lógica multiprofissional, limitações de espaço físico, rotatividade de profissionais e interferência de gestores e coordenadores nas atividades planejadas.

*A gerência do serviço acaba demandando mais para uma categoria profissional e algumas profissões acabam não sendo vistas nos processos de discussão, de atendimentos compartilhados e o quanto isso dificulta a inserção de novos profissionais no cuidado. [...] acaba invisibilizando algumas profissões.* (3 PRMSM)

A quantidade elevada de atendimentos realizados sempre coloca os profissionais em um ritmo que caracteriza outras racionalidades e que não vão ao encontro da interprofissionalidade, precarizando assim o trabalho em saúde. Há ainda uma cobrança para que os residentes supram as demandas dos serviços pela falta de profissionais que existem. Por vezes com a compreensão de que os atendimentos compartilhados são menos producentes quando comparados aos individuais.

*A alta demanda do serviço acaba empurrando a gente para uma prática uniprofissional. E a gente tem que dar conta disso de alguma forma e o interprofissional vai ficando de lado.* (7 PRMSM)

*Falta de profissionais em vários campos da nossa prática. Não conseguimos passar por um processo de formação porque ficamos responsáveis pela demanda do serviço. A falta de garantia de espaços democráticos dificulta muito a gente a fazer um trabalho interprofissional porque muitas vezes a gente toma conta da demanda que seria de um profissional ou do serviço.* (14 PRMSF)

Dificuldades relacionadas aos programas de residência figuram ainda nas seguintes colocações: a falta de atividades planejadas com outros programas de residências e a carga horária semanal de 60 horas. Já aquelas pertinentes ao arcabouço teórico citaram: a falta de entendimento dos preceptores, tutores, coordenadores dos PRMS e gestores dos serviços para o planejamento de ações na lógica interprofissional e a falta de compreensão dos papéis das diferentes profissões e dos próprios residentes.

Essa fragilidade reside não só na dificuldade de se compreender o papel do residente dentro do serviço, mas também na falta de familiaridade com o núcleo profissional de algumas categorias profissionais que fragiliza ainda mais o processo de formação voltado à interprofissionalidade e na inserção de alguns residentes nos cenários de prática. À essa questão, acrescenta-se o desconhecimento por parte dos professores, tutores e preceptores acerca da operacionalização da EIP.

*Não conhecimento sobre o que fazem os diferentes núcleos de saber dificulta a conformação do trabalho. [...] há estigmas e saberes cristalizados sobre o que determinados profissionais fazem.* (1 PRMSM)

*O desconhecimento dos preceptores e coordenadores e mesmo da Coordenação a respeito da prática interprofissional e a importância dela faz com que não tenhamos atividades interprofissionais na Residência de forma planejada.* (12 PRMSF)

Outros desafios foram colocados como a falta de remuneração de tutores e preceptores, a formação uniprofissional nas graduações em saúde, o envolvimento dos profissionais dos serviços nas atividades dos programas de residência e o processo avaliativo frágil.

## DISCUSSÃO

Ao compreender as vivências de residentes em PRMS quanto à EIP e PCI, observou-se que, embora existam iniciativas que favorecem a formação e o trabalho colaborativo, ainda persistem desafios estruturais, organizacionais e pedagógicos que limitam a consolidação da interprofissionalidade. Os achados evidenciaram que a interprofissionalidade é produzida no cotidiano dos serviços, nas relações e práticas desenvolvidas nos cenários de formação, sendo atravessada pelas formas de organização do trabalho, pelas interações entre os sujeitos e pelas condições institucionais presentes nos serviços de saúde. Desse modo, os espaços coletivos identificados pelos residentes favorecem aprendizagens construídas na experiência compartilhada do trabalho em saúde, permitindo que a colaboração interprofissional seja produzida a partir da interação contínua entre diferentes sujeitos, saberes e práticas profissionais.

Os programas de residências representam espaços privilegiados para vivências colaborativas entre as diferentes profissões da saúde, como nos atendimentos compartilhados, nas reuniões de equipe, na tutoria e nas atividades em rede. Enquanto eixo formativo, a EIP potencializa o desenvolvimento de competências tanto individuais quanto coletivas. No entanto, sua efetiva implementação ainda enfrenta lacunas na compreensão conceitual, exigindo suporte institucional contínuo, infraestrutura adequada, domínio de metodologias ativas e mudanças estruturais e culturais no campo da educação na saúde de forma a promover a interação entre os estudantes de diferentes cursos, visando romper com o paradigma do modelo curricular, hegemonicamente uniprofissional e disciplinar^(21,22)^.

A EIP depende de intencionalidade didática declarada, estratégias estruturadas e formação dos tutores e preceptores para que possa ser efetivamente implementada^(13)^, por isso é emergente processos de educação permanente frequentes alinhados às diretrizes e bases dos programas que contribuam para a formação em saúde. Apesar das oportunidades identificadas, os participantes destacaram a ausência de planejamento pedagógico explícito voltado à interprofissionalidade devido à falta de preparo dos participantes (preceptores, tutores, supervisores e docentes), fragilizando as ações interprofissionais.

Desse modo, o planejamento das atividades de ensino e a avaliação do processo de ensino-aprendizagem são partes cruciais e relevantes para a adoção da EIP. Para isso é preciso carga horária declarada nos projetos pedagógicos para a realização de encontros planejados entre tutores, preceptores e residentes visto que asseguram a intencionalidade das atividades e reconhecem a importância da avaliação sistemática e contínua do aprendizado e da aquisição de competências colaborativas, que contemplam a capacidade do estudante atuar em conjunto com outros profissionais, usuários, famílias e comunidades^(23)^.

Nesse sentido, a baixa interação entre tutores, preceptores e residentes pode constituir também um limitador para a compreensão da interprofissionalidade pelos residentes, pois os encontros entre esses poderiam ser norteados pela EIP e PCI, o que nos programas analisados ocorrem de maneira pouco explorados. Assim, as experiências colaborativas desenvolvidas na residência mostram-se diretamente relacionadas à qualidade das interações produzidas no cotidiano do trabalho em saúde e às possibilidades de construção compartilhada do cuidado entre os diferentes sujeitos envolvidos.

O aprendizado em pequenos grupos com a representação equilibrada de todas as categorias profissionais envolvidas, apresenta-se como outra estratégia para facilitar a aprendizagem interprofissional^(23)^, algo percebido apenas no PRMSM por ter número equânime de vagas por categoria profissional. Este achado aponta que a configuração dos cenários de prática tem impacto direto na materialização da interprofissionalidade.

As práticas interprofissionais permitiram aos residentes reconhecerem os saberes dos colegas, refletirem criticamente sobre o cuidado e fortalecerem o trabalho em equipe, promovendo uma formação integrada e centrada nas necessidades do usuário. Tais aprendizagens ultrapassam a dimensão técnico-operacional, envolvendo processos relacionais e comunicacionais construídos nas experiências compartilhadas do cuidado e do trabalho em saúde. Desse modo, a EIP e PCI configuram-se como abordagens promissoras para qualificar o cuidado integral ao desenvolver competências comuns, específicas e colaborativas, complementando e não em substituição aos modelos multiprofissional, multidisciplinar, interdisciplinar ou mesmo uniprofissional^(24)^.

Os PRMS representam uma oportunidade para romper estereótipos profissionais, como evidenciado nos encontros, destacando a importância da clareza dos papéis para um trabalho colaborativo eficaz e a reconstrução dos saberes específicos de cada profissão. O reconhecimento da interdependência entre as áreas reforça a relevância da comunicação interprofissional como competência essencial para superar barreiras, fortalecer relações e qualificar o cuidado por meio de decisões compartilhadas^(25)^.

O desconhecimento sobre o papel de algumas profissões apareceu no discurso dos participantes. Algumas categorias por serem historicamente mais antigas são mais bem compreendidas, mesmo que de forma estereotipada pelos diversos setores da sociedade, como a enfermagem e a medicina. Outras como a terapia ocupacional, por exemplo, geram estranheza entre as demais categorias profissionais e os usuários acerca do seu papel profissional^(26)^.

Algumas categorias profissionais, que compõem os programas aqui analisados, aparecem na literatura como sendo aquelas que mais apresentam dificuldade de inserção nos espaços dos serviços, como a fonoaudiologia, nutrição, psicologia e a terapia ocupacional. Essa situação é agravada pela expectativa colocada nesses profissionais, principalmente aqueles que têm atuação em serviços especializados de forma individualizada, o que contrasta com a lógica interprofissional, necessitando maior compreensão dos papéis e alinhamento dos objetivos para uma integração efetiva dessas categorias nas práticas de saúde^(27)^.

Há diferentes percepções em relação à existência de áreas profissionais mais abertas e disponíveis à EIP. Existem instrumentos, como o *Readiness for interprofessional Learning Scale* (RIPLS) que mensura a prontidão dos estudantes para a aprendizagem compartilhada com diferentes categorias profissionais, avaliando três fatores: o trabalho em equipe e colaboração, a identidade profissional e a atenção à saúde centrada no paciente^(28)^. Todavia, há divergências na literatura entre as escalas utilizadas nos estudos, o que fragiliza a comparação entre os achados que analisam os estudantes e profissionais de forma longitudinal durante e após a realização de uma intervenção^(23,29)^.

Em pesquisa que objetivou conhecer as experiências vivenciadas por egressos de um PRMSF, identificou-se que o programa de residência se apresenta como cenário para a aprender com o outro e compreender o papel do outro^(14,30)^. Já um estudo que analisou a integração dos PRMS à conformação das RAS, constatou-se que esses programas favorecem a operacionalização do trabalho em rede, evidenciando sua importância para a formação profissional^(30)^.

Os residentes presentes no cotidiano dos cenários de prática resultam em um aumento na força de trabalho dos serviços e na capacidade da equipe solucionar os problemas de saúde dos usuários ao mesmo tempo em que gera uma confusão no entendimento de quais seriam os seus papéis nos cenários de formação, sendo tomados como possibilidade de suprir a falta de força de trabalho em saúde, desviando da premissa dos PRMS^(30)^.

A essa questão, somam-se outros fatores que impactam no ensino-aprendizagem dos PRMS que incluem a divergência entre a proposta pedagógica e sua aplicação prática, desconexão entre currículo e as atividades de campo, conflitos interpessoais, estereótipos, falta de reconhecimento dos papéis, resistência à EIP, modelos tradicionais de formação, pouca capacitação dos tutores, supervisão limitada, comunicação ineficaz e a valorização do desempenho individual e da competitividade^(11,30)^. Esses condicionantes demonstram que a consolidação da interprofissionalidade depende não apenas da disposição individual dos profissionais e residentes, mas também das condições organizacionais, pedagógicas e institucionais que estruturam os processos de trabalho e formação em saúde. Para mudar essa realidade, é necessário um esforço conjunto das instituições formadoras, serviços de saúde e políticas públicas de saúde e educação que valorizem a EIP como base da formação em saúde.

Os limitadores desta pesquisa decorrem da investigação ter ocorrido em apenas uma instituição proponente de PRMS, assim, como, a não participação de preceptores, tutores, coordenadores, usuários e profissionais dos serviços, o que impossibilita a análise do contexto a partir da vivência de todos os sujeitos envolvidos nos cenários de prática e formação dos programas de residência. Soma-se a isso o fato de a coleta de dados ter ocorrido por meio de encontros remotos e síncronos, o que pode ter influenciado a interação entre pesquisadores e participantes, bem como a apreensão de elementos não verbais da comunicação.

Os achados deste estudo evidenciam avanços relevantes para a enfermagem e para a área da saúde ao reafirmarem os PRMS como cenários estratégicos para o desenvolvimento da educação e da prática colaborativa interprofissional. Ao evidenciar aprendizagens relacionadas à comunicação interprofissional, ao reconhecimento de papéis e à tomada de decisão compartilhada, o estudo contribui para o fortalecimento de competências essenciais para o cuidado integral no SUS. Para o campo da saúde, os achados ampliam a compreensão sobre os condicionantes pedagógicos, organizacionais e institucionais que favorecem ou limitam a interprofissionalidade nos cenários de formação em serviço, oferecendo subsídios para o aprimoramento de projetos pedagógicos, políticas de educação permanente e modelos de gestão do trabalho em saúde.

Adicionalmente, estudos que analisam processos formativos e dinâmicas interprofissionais em residências apresentam caráter estruturante e não meramente circunstancial, uma vez que abordam dimensões organizacionais, pedagógicas e relacionais que tendem a se manter ao longo do tempo, mesmo diante de mudanças conjunturais. Assim, os resultados produzidos com essa pesquisa permanecem atuais e relevantes, contribuindo para o avanço do conhecimento na área, subsidiando a qualificação de programas de residência e apoiando a formulação e o aprimoramento de políticas públicas voltadas à formação em saúde.

## CONSIDERAÇÕES FINAIS

A presente pesquisa permitiu conhecer as vivências de educação e prática colaborativa interprofissional de residentes em programas de residência multiprofissional em saúde, evidenciando oportunidades formativas, aprendizagens desenvolvidas, componentes necessários e desafios para a consecução da interprofissionalidade nos cenários de formação em serviço. Os achados mostraram que essas vivências se materializam principalmente em espaços cotidianos de interação entre residentes e equipes dos serviços, como atendimentos compartilhados, discussões de caso e reuniões de equipe, favorecendo o reconhecimento dos papéis profissionais e o desenvolvimento de competências colaborativas.

Contudo, os resultados também evidenciaram que a consolidação da interprofissionalidade ainda é tensionada por aspectos estruturais, organizacionais e pedagógicos dos serviços e programas de residência, como a predominância da lógica multiprofissional de formação e trabalho em saúde, a elevada demanda por atendimentos nos serviços e a ausência de planejamento intencional de atividades interprofissionais. Dessa forma, é imperativo que a EIP seja implementada nos diferentes processos formativos, rompendo com paradigmas tradicionais de ensino e do trabalho em saúde, com vistas a melhorar o cuidado em saúde e a colaboração entre os profissionais.

Vislumbra-se, por fim, a importância de pesquisas que considerem diferentes contextos, abordagens e populações, especialmente estudos longitudinais, intervencionistas e de métodos mistos, ainda escassos na temática da educação e prática interprofissional. Acredita-se que novas investigações poderão subsidiar mudanças na formação e na formulação de novas políticas públicas de saúde e educação.

## Data Availability

Todo o conjunto de dados que dá suporte aos resultados deste estudo foi publicado no próprio artigo.

# Translation: Experiencias de educación y práctica colaborativa interprofesional en residencias multiprofesionales en salud*

**DOI:** 10.1590/1980-220X-REEUSP-2025-0588es

**Authors:** Lucas Cardoso dos Santos, Thiago da Silva Domingos, Eduardo Gabriel Cassola, Valéria Marli Leonello, Carolyne Reduzina Queirós, Marcelo Viana da Costa, Wilza Carla Spiri

**Affiliations:** 1 Universidade de São Paulo, Escola de Enfermagem, Departamento de Enfermagem em Saúde Coletiva, São Paulo, SP, Brasil.; 2 Universidade Estadual Paulista “Júlio de Mesquita Filho”, Faculdade de Medicina de Botucatu, Departamento de Enfermagem, Botucatu, SP, Brasil.; 3 Universidade de São Paulo, Escola de Enfermagem, Departamento de Orientação Profissional, São Paulo, SP, Brasil.; 4 Universidade Federal do Rio Grande do Norte, Escola Multicampi de Ciências Médicas, Caicó, RN, Brasil.

**Keywords:** Educación, Educación Interprofesional, Especialización, Relaciones Interprofesionales

## Abstract

**Objetivo:**

Conocer las experiencias de educación y práctica colaborativa interprofesional de los residentes de programas de residencia multiprofesional en salud.

**Método:**

Estudio descriptivo, exploratorio, con enfoque cualitativo. Participaron 18 residentes vinculados a dos programas de residencia de una universidad pública brasileña del estado de São Paulo. La recolección de datos se llevó a cabo mediante dos reuniones remotas sincrónicas. El análisis del material empírico se realizó mediante el análisis de contenido.

**Resultados:**

Los temas pusieron de manifiesto las oportunidades para la educación y la práctica interprofesional, como las consultas compartidas, y la necesidad de querer conocer al otro y trabajar con él. Se pusieron de manifiesto las dificultades que enfrentaron los residentes en relación con los procesos de trabajo en salud de los servicios, al operar bajo una lógica multiprofesional.

**Consideraciones finales:**

Las experiencias de educación y práctica colaborativa interprofesional en las residencias multiprofesionales se dieron principalmente en espacios compartidos de atención y aprendizaje, lo que favoreció el desarrollo de competencias colaborativas. Sin embargo, los desafíos organizacionales, pedagógicos y relacionados con los procesos de trabajo en salud aún limitan la consolidación de la interprofesionalidad en la formación y el trabajo.

## INTRODUCCIÓN

La Educación Interprofesional (EIP) se da cuando los miembros de más de una profesión de la salud aprenden juntos, de manera integrada e interactiva, con el propósito explícito de mejorar la colaboración interprofesional y/o la atención en salud de los usuarios, las familias y las comunidades^(1)^. Se presenta como un enfoque educativo innovador y potente para la formación del personal de salud, ya que permite el desarrollo de competencias colaborativas, como la comunicación interprofesional, la atención centrada en el usuario, la familia y la comunidad, la comprensión de los roles profesionales y la dinámica organizativa del equipo, la resolución de conflictos interprofesionales y el liderazgo colaborativo^(1,2)^.

La EIP proporciona una Práctica Colaborativa Interprofesional (PCI), entendida como la colaboración en la práctica entre miembros de dos o más profesiones que actúan de manera interactiva, compartiendo objetivos y reconociendo el papel y la importancia del otro en la complementariedad de las acciones en salud^(1)^. La literatura evidencia que la PCI fortalece los servicios de salud a partir de la mejora en la calidad de la atención de salud ofrecida, una mayor satisfacción del usuario, el compromiso de los profesionales involucrados con la seguridad del paciente, la reducción de errores y la duplicación de procedimientos, la superación de estereotipos y la falta de conocimiento entre las profesiones sobre las demás áreas profesionales, el intercambio de conocimientos para la toma de decisiones y una mayor participación de los usuarios en la planificación de la atención^(1,2,3,4,5)^.

La interprofesionalidad constituye un marco teórico-conceptual y metodológico que orienta la formación y el trabajo en salud, con el objetivo de brindar una atención integral y centrada en las necesidades de los usuarios, las familias y las comunidades. Se basa en la comprensión de que el trabajo interprofesional abarca diferentes modalidades de interacción entre profesiones —como el trabajo en equipo, la colaboración, la práctica colaborativa y el trabajo en red— y no es sinónimo de trabajo en equipo. Estas modalidades se diferencian por el grado de articulación de las acciones y de interacción entre los agentes, expresado en el intercambio de valores, objetivos e identidad^(6)^.

En este espectro, el trabajo en equipo constituye la dimensión más central del trabajo interprofesional, ya que implica una mayor interdependencia, integración y responsabilidad conjunta por la atención, siendo fundamental para responder a la complejidad de las necesidades de salud. Por su parte, la EIP implica un aprendizaje interactivo e intencional entre diferentes profesiones, lo que favorece el desarrollo de prácticas colaborativas interprofesionales y mejores resultados en salud. Metodológicamente, supone estrategias de enseñanza que promuevan la interacción, el diálogo y la construcción compartida, contribuyendo al desarrollo de competencias colaborativas y a la mejora de la calidad de la atención y de los procesos de trabajo en salud^(1,2,3,4,5,6)^.

A nivel mundial, la EIP surgió en el escenario internacional en las décadas de 1960 y 1970, a partir de iniciativas de formación conjunta entre diferentes profesiones de la salud, especialmente en países como el Reino Unido y Canadá, y posteriormente se fortaleció como agenda global con la publicación del informe de la Organización Mundial de la Salud en 2010. Este hito contribuyó a difundir la propuesta del aprendizaje interactivo entre profesiones como estrategia para fortalecer los sistemas de salud, en especial la atención primaria. A partir de este movimiento, diversas experiencias internacionales comenzaron a poner de manifiesto el potencial de la EIP para mejorar la calidad de la atención, cuestionar los modelos de formación fragmentados y promover prácticas colaborativas, consolidándose como un enfoque relevante para superar la lógica de los silos profesionales en la formación y el trabajo en el ámbito de la salud^(1)^.

En Brasil, el Sistema Único de Salud (SUS), guiado por el principio de integralidad, requiere la formación de equipos interprofesionales capaces de responder de manera integral y coordinada a las necesidades de salud de la población^(7)^. En este contexto, las experiencias de EIP en el ámbito nacional, aunque aún incipientes, se han visto fortalecidas por la labor de la Red Brasileña de Educación y Trabajo Interprofesional en Salud (ReBETIS) y por políticas que impulsan cambios en la formación, como el Programa de Educación a través del Trabajo para la Salud (PET-Saúde) y los Programas de Residencias Multiprofesionales en Salud (PRMS), que amplían la integración entre profesiones, fortalecen la práctica colaborativa y contribuyen a la reorientación de la formación en salud en el país^(8)^.

Los PRMS, regulados en 2005 en Brasil, son ejemplos de la modalidad de educación de posgrado *lato sensu*, orientada a diversas profesiones de la salud —biomedicina, ciencias biológicas, educación física, enfermería, farmacia, física médica, fisioterapia, fonoaudiología, medicina veterinaria, nutrición, odontología, psicología, trabajo social, terapia ocupacional y salud colectiva—, con excepción de la medicina^(9,10)^.

Los PRMS tienen como objetivo formar profesionales capaces de trabajar en equipo, integrando conocimientos y promoviendo una atención integral alineada con las necesidades de salud^(9,10)^. Contribuyen a la reformulación del modelo de atención de salud al buscar estándares más humanizados y adecuados para satisfacer las necesidades de la población^(11,12)^. Ofrecen oportunidades de trabajo interprofesional, orientado por la EIP, lo que permite una práctica colaborativa con mejores resultados en salud^(12)^.

Los PRMS representan un ámbito estratégico para el desarrollo de la EIP y la PCI, ya que contribuyen directamente a la formación de profesionales mejor preparados para enfrentar los desafíos de la salud pública con ética y compromiso, tomando como punto de partida las necesidades de salud de los usuarios, las familias y las comunidades^(11 12,13,14)^. En este sentido, las residencias multiprofesionales constituyen una política pública que impulsa la reorientación de la formación en salud, asumiendo un papel central en la consolidación de las prácticas interprofesionales en el SUS^(9)^. Sin embargo, a pesar de los reconocidos beneficios, la EIP y la PCI, en el contexto de los PRMS, aún se exploran muy poco en la literatura^(12,13)^, lo que pone de manifiesto la necesidad de ampliar la producción científica sobre la interprofesionalidad en este ámbito.

De este modo, incorporar la EIP en los diferentes planes de estudio y entornos de formación de los posgrados en salud se configura como un enfoque potente para romper los estereotipos de las profesiones y la forma de hacer y pensar la salud^(11,12,13,14)^, en lo que los PRMS se presentan como una posibilidad para la enseñanza y la práctica interprofesional. Por lo tanto, la pregunta central de esta investigación es: ¿son los Programas de Residencia Multiprofesional en Salud espacios potentes para el desarrollo de la educación y la práctica colaborativa interprofesional? Para ello, se buscó conocer las experiencias de educación y práctica colaborativa interprofesional de los residentes de programas de residencia multiprofesional en salud.

## MÉTODO

### Tipo de Estudio

Se trata de una investigación descriptiva, exploratoria y de naturaleza cualitativa^(15)^ que tomó en cuenta las recomendaciones de los *Criterios Consolidados para el Informe de Investigación Cualitativa* ^(16)^.

### Lugar, Población y Criterios de Selección

El escenario de la investigación fue una institución de educación superior pública, ubicada en un municipio del centro-oeste del estado de São Paulo, Brasil, con una población estimada de 149 718 habitantes. Ofrece cursos de pregrado en enfermería y medicina, así como programas de posgrado en las modalidades *stricto sensu* y *lato sensu*, como los Programas de Residencia Multiprofesional en Salud Familiar (PRMSF) y Salud Mental (PRMSM), a los que se ingresa mediante un proceso de selección anual. Estos programas tienen una duración de dos años y cuentan con una carga horaria semanal de 60 horas y un total de 5 760 horas, distribuidas entre actividades prácticas desarrolladas en los servicios de salud y actividades teóricas y teórico-prácticas realizadas en la institución formadora.

Dichas actividades incluyen momentos formativos específicos por área profesional y espacios compartidos entre diferentes profesiones, orientados al análisis de casos, la planificación de la atención y la reflexión sobre el proceso de trabajo en salud, lo que favorece las experiencias de educación y práctica interprofesional. La elección de este escenario se justificó por tratarse de programas consolidados, vinculados a una institución pública reconocida por la integración entre universidad, servicio y comunidad.

Los participantes fueron residentes vinculados a estos programas, seleccionados por estar directamente involucrados en procesos formativos que exigen la interacción entre diferentes profesiones y el trabajo en equipo, por lo que se les consideró informantes clave para comprender las experiencias relacionadas con la educación y la práctica interprofesional en el contexto de la residencia multiprofesional.

El PRMSF está compuesto por las categorías profesionales de educación física, enfermería, fisioterapia, nutrición, odontología, psicología y trabajo social, con cuatro cupos para el curso de enfermería y un cupo para las demás, lo que suma un total de diez residentes que ingresan por año. Por su parte, el PRMSM incluye enfermería, psicología, trabajo social y terapia ocupacional, con dos cupos por categoría al año, lo que totaliza ocho residentes.

Los entornos de trabajo de los residentes varían entre los diferentes puntos de las Redes de Atención a la Salud (RAS), como las Unidades Básicas de Salud, los Centros de Salud Escolar y los Servicios de Atención Psicosocial Estratégica, Desinstitucionalización y Rehabilitación Psicosocial, entre otros. En los programas participan coordinadores, vicecoordinadores, preceptores, tutores y docentes, quienes no reciben una *remuneración* por el desempeño de estas funciones. Algunos servicios, al recibir residentes de más de un programa de la institución, cuentan con profesionales que desempeñan funciones superpuestas; es decir, a veces actúan como preceptores y otras como tutores.

Los participantes elegibles para la investigación fueron los residentes con matrículas activas en el segundo año de los programas PRMSF y PRMSM que mostraban interés en colaborar con la investigación. Por su parte, los criterios de exclusión fueron: baja de la residencia; licencia médica prolongada o período de vacaciones que coincidiera con la etapa de recolección de datos. Todos los residentes de dichos programas aceptaron participar en la investigación, lo que sumó un total de 18 participantes; por lo tanto, no hubo rechazos.

### Recolección de Datos

La recolección de datos se llevó a cabo mediante un taller participativo estructurado en dos sesiones remotas sincrónicas, con el uso de dinámicas grupales y discusión colectiva mediada, enfocadas en la reflexión sobre las experiencias y la construcción de reflexiones y significados entre los participantes^(17)^.

Se planificó junto con las coordinaciones del PRMSF y el PRMSM para que se llevara a cabo dentro del horario de las actividades académicas de los residentes, con el fin de garantizar una mayor participación de los participantes, sin afectar las actividades planificadas por los programas de residencia. También se consideró la realización de la actividad en períodos en los que los residentes tuvieran actividades curriculares en común. Se llevaron a cabo dos encuentros remotos sincrónicos programados a través de un sistema de videoconferencia (*Google Meet*), los cuales fueron grabados, con un intervalo de una semana entre ellos y una duración aproximada de cuatro horas cada uno, en el mes de junio de 2021, en medio de la pandemia de COVID-19.

La primera sesión comenzó con la bienvenida a los participantes por parte del primer autor, enfermero y maestro en salud pública. Posteriormente, se explicaron los objetivos de la investigación, se aclararon posibles dudas y se ofreció un formulario electrónico, elaborado en *Google Forms,* para el registro del consentimiento libre e informado, de acuerdo con las directrices de la Comisión Nacional de Ética en Investigación^(18)^, así como un cuestionario autoadministrado para la caracterización sociodemográfica, que incluía: género, edad, título universitario (año de graduación; nombre, ciudad y estado de la institución formadora; modalidad del curso —presencial, semipresencial o a distancia—) y el programa de residencia al que estaban vinculados.

A continuación, se dividió a los participantes por programa de residencia en dos salas virtuales distintas, con la coordinación de un mediador por grupo: un enfermero con doctorado en enfermería y una enfermera con maestría en enfermería. El investigador principal se desplazó entre ambos espacios para aclarar posibles dudas y seguir las discusiones. Cabe destacar que tanto los moderadores como el investigador cuentan con experiencia previa en el desarrollo de investigaciones cualitativas y no tenían relaciones previas ni cercanas con los participantes de la investigación.

Los objetivos del primer encuentro fueron: recabar los conocimientos de los participantes sobre la EIP y la PCI, y conocer el proceso de enseñanza-aprendizaje-trabajo en ambos programas. Se utilizaron preguntas de lanzamiento para que los residentes debatieran en grupo, con una presentación posterior en sesión plenaria ante todos los participantes del taller: (1) ¿En qué situaciones de las actividades de la residencia tienes la oportunidad de actuar de manera interprofesional? (2) ¿Qué caracteriza a estas actividades como interprofesionales?; (3) En tu opinión, ¿qué facilita la práctica del trabajo interprofesional en el contexto de las actividades de la residencia?; (4) En tu opinión, ¿qué dificulta la práctica del trabajo interprofesional en el contexto de las actividades de la residencia?».

Cada grupo debatió y validó colectivamente los puntos planteados a partir de cada pregunta orientadora. Las intervenciones de los participantes se complementaron entre sí, sin que se observaran divergencias manifiestas entre los residentes durante las discusiones grupales por programa de residencia. De esta manera, los contenidos generados por cada grupo representaron consensos construidos colectivamente por los participantes de un mismo programa. Posteriormente, en sesión plenaria, un representante de cada grupo presentó una propuesta de síntesis, la cual fue nuevamente discutida y validada colectivamente por los participantes de ambos programas.

La segunda reunión se llevó a cabo con el investigador principal, quien, en primer lugar, retomó los objetivos de la investigación, la propuesta de síntesis elaborada por los grupos en la primera reunión y presentó la propuesta de trabajo para la segunda reunión. A continuación, se realizó una presentación interactiva sobre el marco teórico-conceptual y metodológico de la EIP y la PCI a cargo de un especialista en la materia, enfermero y doctor en ciencias de la salud.

Posteriormente, los residentes se dividieron nuevamente en dos grupos por programa, en salas virtuales distintas, en las que debían revisar la síntesis elaborada a partir de nuevas preguntas orientadoras: (1) ¿Cuáles fueron los aprendizajes más destacados de estas experiencias? ¿Fueron estimulados o desarrollados de diferentes maneras por los residentes?; (2) ¿Cuál(es) fue(ron) la(s) intencionalidad(es) más explícita(s) de estas iniciativas? ¿Cómo se expresó esa intencionalidad?; (3) ¿Qué etapas del proceso de enseñanza-aprendizaje facilitaron el desarrollo de competencias para el trabajo eficaz en equipo?; (4) ¿En qué momento se discutieron las facilidades y/o dificultades para la evaluación y la planificación del proceso de enseñanza-aprendizaje?

De esta manera, intencionalmente, se revisaron las respuestas dadas en la primera sesión a partir de nuevas preguntas, con el objetivo de generar un debate en el grupo a partir del marco teórico-conceptual y metodológico de la EIP presentado. Después de este momento, cada grupo presentó en plenaria una nueva síntesis de los puntos debatidos.

Al final del taller, se proporcionó un enlace para acceder al formulario de evaluación de la actividad. La primera parte contenía una escala tipo *Likert* con cinco criterios (no estoy de acuerdo en absoluto, no estoy de acuerdo en parte, neutral, estoy de acuerdo en parte, estoy de acuerdo totalmente) relacionados con los objetivos del taller, la organización del taller, la actuación del investigador principal, el invitado y los mediadores, y la autoevaluación de los participantes. En la segunda parte, los participantes debían completar las frases que comenzaban con las expresiones: «Ni siquiera lo esperaba y sucedió...», «Me decepcionó un poco...», «Si tuviera que promocionar este taller ante un colega, le diría que...».

### Análisis y Tratamiento de los Datos

Los datos de la investigación se organizaron según el análisis de contenido propuesto por Graneheim et al.^(19)^ en su vertiente cualitativa. La aplicación de este marco de análisis se llevó a cabo a partir de la lectura del texto, representado por la transcripción de las grabaciones de las dos sesiones y las anotaciones realizadas por los mediadores con la intención de extraer y reunir un nuevo documento que constituyera la unidad de análisis. Posteriormente, se identificaron unidades de significado, entendidas como palabras, frases o párrafos, que contenían aspectos relacionados entre sí a través de su contenido y contexto. Estas unidades se condensaron, conservando sus núcleos de significado, y posteriormente se codificaron. Los códigos con aproximación temática se agruparon, dando lugar a cuatro temas (Cuadro 1). Para el análisis de los datos se tomó como referencia el marco de la interprofesionalidad^(1,2,3,4,5,6)^.

**Cuadro 1:** Presentación del marco de referencia, los temas, las unidades de significado y la representación de los participantes – Botucatu, SP, Brasil, 2021.

Referencial	Temas	Unidades de Significado*	Representación de los PRMS
Interprofesionalidad	Oportunidades identificadas como prometedoras para la enseñanza y la práctica interprofesional	Atenciones compartidas; Planificación y ejecución de actividades comunes; Debates y estudios de caso en el modelo de apoyo matricial; Reuniones del equipo del servicio; Tutoría; Trabajo en red e intersectorial	PRMSM PRMSF
		Estrategia de enseñanza; Encuentros espontáneos e informales; Trabajo en grupos con todas las categorías del PRMSM	PRMSM
		Reuniones entre los residentes y la coordinación del PRMSF; Elaboración: genograma, ecomapa, proyecto terapéutico individual; Clases teóricas con otros programas	PRMSF
	Aprendizajes desarrollados a través de la práctica compartida	Nuevas formas de abordar al usuario; Identificación de las necesidades de salud; Desarrollo de las competencias interprofesionales; Dinámica del trabajo colaborativo; Resultados de la EIP y la PCI	PRMSM
	Componentes necesarios para la enseñanza y la práctica interprofesional	Disponibilidad para la EIP y la PCI; Conocimiento de los roles profesionales; Vínculo entre los residentes; Conocimiento sobre la EIP y la PCI por parte de los tutores, preceptores y la coordinación; Intencionalidad para la EIP y la PCI	PRMSM PRMSF
	Desafíos para la implementación de la enseñanza y la práctica interprofesional	Complejidad de las necesidades de salud; Procesos de trabajo de los servicios; Resistencia al cambio; Infraestructura; Rotación de profesionales; Interferencia de los gestores municipales y coordinadores de los programas; Organización de las actividades de los programas	PRMSM PRMSF

*Las unidades de significado presentadas corresponden a los consensos grupales elaborados y validados por los residentes de cada programa.

### Aspectos Éticos

La presente investigación fue aprobada con el número de dictamen 3.961.371 el 2 de marzo de 2020 por el Comité de Ética en Investigación de la institución proponente y siguió todas las recomendaciones relacionadas con las investigaciones que involucran a seres humanos y cualquier etapa en un entorno virtual^(18,20)^. Para utilizar extractos de las intervenciones de los participantes en el taller, se empleó un código alfanumérico compuesto por un dígito seguido de la sigla del programa (PRMSM, PRMSF), suprimiendo cualquier información que pudiera identificarlos, como la categoría profesional y el nombre de los tutores, preceptores y coordinadores.

## RESULTADOS

La edad de los participantes osciló entre los 22 y los 34 años, con una mayor representación de aquellos de 25 años; 12 personas se identificaron como mujeres y seis como hombres. Todos completaron la licenciatura en modalidad presencial en instituciones del estado de São Paulo, 16 de ellos en instituciones públicas y, en su mayoría, en el año 2019.

La evaluación del taller se consideró positiva, ya que los aspectos relacionados con los objetivos y la organización del taller, la actuación del investigador principal y de los mediadores, así como la autoevaluación de los residentes, se analizaron en su gran mayoría con el criterio «estoy totalmente de acuerdo».

Las reflexiones derivadas de las preguntas abiertas confirmaron los relatos iniciales, mostrando que los participantes reflexionaron sobre la práctica, la formación durante la residencia y la EIP y la PCI, además de intercambiar experiencias. A pesar de las frustraciones con la realidad y la percepción de que la interprofesionalidad aún está lejos de la práctica, reconocieron la importancia del taller para evaluar el proceso de enseñanza-aprendizaje-trabajo, compartir dificultades y reflexionar sobre nuevas formas de trabajo en equipo y atención en salud.

Los resultados del análisis del material empírico proveniente de los dos encuentros permitieron identificar unidades de significado, que posteriormente se condensaron y agruparon en cuatro temas: Oportunidades identificadas como potentes para la educación y la práctica interprofesional; Aprendizajes desarrollados a través de la práctica compartida; Componentes necesarios para la educación y la práctica interprofesional; Desafíos para la consecución de la educación y la práctica interprofesional.

### Oportunidades Identificadas como Prometedoras Para la Educación y la Práctica Interprofesional

El primer tema aborda las oportunidades identificadas en las actividades de la residencia que se consideran prometedoras para una actuación interprofesional. Entre las que figuran en ambos programas, se encuentran: las atenciones compartidas realizadas a través de consultas y visitas a domicilio, la planificación y ejecución de actividades comunes, las discusiones y los estudios de caso en el modelo de apoyo matricial, las reuniones de equipo, la tutoría y el trabajo en red e intersectorial.

*Las atenciones compartidas fueron un tema recurrente en nuestro grupo. Tener la posibilidad de atender junto con el otro, de estar ahí, de discutir esa necesidad, de analizarla con mayor profundidad después, de estar ahí también como apoyo, como respaldo en el momento de la atención.* (3 PRMSM)

*Las reuniones son importantes porque nos permiten revisar los procesos de trabajo [...] facilitan un enfoque más interprofesional en las reuniones de equipo. ¡Esto es importante! Comprender el proceso de trabajo, qué son estas experiencias y qué aporta valor a nuestra formación.* (15 PRMSF)

Las discusiones en el modelo de apoyo matricial se mencionaron como un elemento potente para el trabajo interprofesional, ya que se presentan como un espacio en el que se elabora y debate el pensamiento conjunto, y no solo se tiene la intención de derivar al usuario a otro profesional.

Los residentes del PRMSM mencionaron el uso de la estrategia de enseñanza del Aprendizaje Basado en Problemas y los encuentros espontáneos e informales en el ejercicio del trabajo. Por su parte, los residentes del PRMSF mencionaron situaciones específicas además de las ya citadas: las reuniones entre los residentes y la coordinación del programa, la elaboración del genograma, el ecomapa y el Proyecto Terapéutico Singular (PTS), el uso de instrumentos para el enfoque familiar y las clases teóricas con residentes de otros programas de residencia.

Los participantes del PRMSM coincidieron al mencionar que el hecho de trabajar en los servicios en grupos de residentes con todas las categorías profesionales del programa permite que la EIP se materialice con mayor facilidad, a diferencia del PRMSF, en el que la división de los residentes por los escenarios de práctica se da de tal manera que no permite una característica similar.

*Estar cerca de los pacientes facilita el trabajo. Logramos rotar por los campos de práctica como un mini-equipo. Rotamos por los servicios, pero como un mini-equipo. Y en los espacios de guardia, nos reunimos como un gran grupo de salud mental. Esto facilita nuestro intercambio y, además, permite crear vínculos y espacios de discusión.* (3 PRMSM)

El hecho de estar siempre juntos y en los mismos entornos de práctica se mencionó como una forma de que el residente participe de manera continua en la práctica del otro, comprenda el funcionamiento del grupo, planifique y dé continuidad a las actividades programadas, tome decisiones, resuelva problemas más rápidamente y (re)conozca los roles profesionales.

### Aprendizajes Desarrollados a Través de la Práctica Compartida

El segundo tema aborda los aprendizajes de los residentes en la práctica compartida con profesionales de diferentes categorías, destacando la comprensión de nuevas formas de abordar al usuario, la identificación de las necesidades reales de salud, el reconocimiento de las competencias de los colegas y la dinámica del trabajo colaborativo, ya sea multiprofesional o interprofesional.

*Las consultas compartidas permitieron aprender más sobre la forma de trabajar de los demás profesionales.* (6 PRMSM)

Los residentes destacaron que, con el tiempo, lograron identificar competencias específicas de los profesionales y percibieron mejoras en la calidad de la atención y en los resultados de salud. Sin embargo, esta atención colaborativa ocurre más entre ellos que con los profesionales de los servicios. Las discusiones en equipo, a pesar de los conflictos, promueven el acercamiento, la construcción conjunta de acciones, la reflexión sobre la práctica, la resolución de conflictos y una mejor comprensión del funcionamiento de los equipos.

*Las actividades conjuntas que realizamos también son buenas para resolver los conflictos que existen entre nosotros, incluso en nuestras opiniones diferentes sobre un mismo caso.* (1 PRMSM)

Ambos grupos señalaron que las actividades de los programas preparan a los residentes en y para la práctica, y se destacaron por permitir (re)pensar el trabajo realizado y el desarrollo de competencias colaborativas, aunque sin que los participantes lo mencionaran de manera explícita o declarada.

### Componentes Necesarios Para la Educación y la Práctica Interprofesional

El tercer tema abarca los componentes y las características señaladas por los residentes como elementos inherentes al logro de una práctica interprofesional, tales como el deseo de conocer las necesidades del otro, los roles profesionales de los compañeros y el aprendizaje con el otro y en equipo. Destacaron la necesidad de comprender que no existen conocimientos absolutos y que el profesional debe estar capacitado para colaborar en las lagunas de conocimiento de sus colegas sin emitir juicios. Por lo tanto, es necesario dar un paso atrás para confiar en lo que el otro afirma, aceptando que hay cosas que no se comprenden.

*El otro tiene que entender que su conocimiento, su formación, llegará a un límite y que, ante ese límite, debemos trabajar como equipo para poder ampliar esa atención. Por mi cuenta puedo llegar hasta cierto punto, pero a partir de ahí, ¿cómo voy a brindar esa atención?* (3 PRMSM)

Por otro lado, ambos grupos mencionaron el vínculo como una característica que facilita las interacciones y que permite expresar las inseguridades y las lagunas de conocimiento sin temor a ser juzgado por el colega, lo que hace posible un aprendizaje colaborativo. La disposición personal a colaborar, la capacidad de escuchar, el respeto entre los profesionales, la confianza en el trabajo y en los conocimientos del colega también fueron características mencionadas por los residentes.

*El otro debe estar disponible para que podamos cuestionar, conversar y discutir los temas que surgen en la práctica. La disponibilidad es un factor muy importante. Sobre todo, la de saber escuchar.* (3 PRMSM)

Los participantes de ambos programas destacaron como atributos diferenciadores que los preceptores, tutores, coordinadores y gestores de los servicios tuvieran conocimientos sobre la EIP y la PCI. La falta de estos conocimientos se considera una justificación para que algunos entornos sean percibidos como malos campos de práctica por parte de los residentes.

*Cuando hay intencionalidad e interés por hacer de las prácticas algo interprofesional, las cosas en las residencias suceden de manera diferente, pero no vemos una planificación de las actividades para que sean interprofesionales de manera explícita. (9 PRMSF)*

*Existe una dominación por parte de las exigencias del trabajo que dificulta la intencionalidad. La intencionalidad queda subordinada a la demanda.* (3 PRMSM)

Se puso de manifiesto la necesidad de que exista una intencionalidad declarada en la planificación de las actividades interprofesionales por parte de los sujetos involucrados en la residencia; sin embargo, en la práctica se da una inversión de esta lógica que termina centrándose en las demandas de atención de los servicios y no en el desarrollo de las competencias colaborativas.

### Desafíos Para la Consecución de la Educación y la Práctica Interprofesional

El cuarto tema aborda las dificultades que enfrentan los residentes en la práctica interprofesional, destacando la complejidad de las necesidades de salud de los usuarios, la falta de tiempo para el diálogo, la hegemonía de algunas categorías profesionales, la resistencia al cambio, el predominio de la lógica multiprofesional, las limitaciones del espacio físico, la rotación de profesionales y la interferencia de los administradores y coordinadores en las actividades planificadas.

*La gerencia del servicio termina exigiendo más a una categoría profesional y algunas profesiones terminan quedando fuera de los procesos de discusión y de la atención compartida, lo que dificulta la incorporación de nuevos profesionales a la atención. [...] termina invisibilizando a algunas profesiones.* (3 PRMSM)

El elevado número de consultas realizadas siempre impone a los profesionales un ritmo que se rige por otras lógicas y que no se ajusta a la interprofesionalidad, lo que precariza así el trabajo en el ámbito de la salud. Además, se exige a los residentes que cubran las demandas de los servicios debido a la falta de profesionales. A veces, con la idea de que las consultas compartidas son menos productivas en comparación con las individuales.

*La alta demanda del servicio termina empujándonos hacia una práctica uniprofesional. Y tenemos que hacer frente a eso de alguna manera, por lo que el enfoque interprofesional queda relegado.* (7 PRMSM)

*Falta de profesionales en varios ámbitos de nuestra práctica. No logramos pasar por un proceso de formación porque nos hacemos cargo de la demanda del servicio. La falta de garantía de espacios democráticos nos dificulta mucho realizar un trabajo interprofesional porque, muchas veces, nos hacemos cargo de la demanda que correspondería a un profesional o al servicio.* (14 PRMSF)

Las dificultades relacionadas con los programas de residencia también figuran en las siguientes observaciones: la falta de actividades planificadas con otros programas de residencia y la carga horaria semanal de 60 horas. En cuanto a las dificultades relacionadas con el marco teórico, se mencionaron: la falta de comprensión por parte de los preceptores, tutores, coordinadores de los PRMS y administradores de los servicios para planificar acciones con un enfoque interprofesional, y la falta de comprensión de los roles de las diferentes profesiones y de los propios residentes.

Esta debilidad radica no solo en la dificultad para comprender el papel del residente dentro del servicio, sino también en la falta de familiaridad con el núcleo profesional de algunas categorías profesionales, lo que debilita aún más el proceso de formación orientado a la interprofesionalidad y la inserción de algunos residentes en los entornos de práctica. A esto se suma el desconocimiento por parte de los profesores, tutores y preceptores sobre la puesta en práctica de la EIP.

*El desconocimiento sobre lo que hacen los diferentes núcleos de conocimiento dificulta la organización del trabajo. [...] existen estigmas y conocimientos cristalizados sobre lo que hacen ciertos profesionales.* (1 PRMSM)

*El desconocimiento de los preceptores y coordinadores, e incluso de la Coordinación, respecto a la práctica interprofesional y su importancia hace que no contemos con actividades interprofesionales en la residencia de manera planificada.* (12 PRMSF)

Se señalaron otros desafíos, como la falta de remuneración de los tutores y preceptores, la formación uniprofesional en los programas de pregrado en salud, la participación de los profesionales de los servicios en las actividades de los programas de residencia y el frágil proceso de evaluación.

## DISCUSIÓN

Al analizar las experiencias de los residentes en el PRMS en relación con la EIP y la PCI, se observó que, si bien existen iniciativas que favorecen la formación y el trabajo colaborativo, aún persisten desafíos estructurales, organizativos y pedagógicos que limitan la consolidación de la interprofesionalidad. Los hallazgos evidenciaron que la interprofesionalidad se genera en el día a día de los servicios, en las relaciones y prácticas desarrolladas en los entornos de formación, y está determinada por las formas de organización del trabajo, las interacciones entre los sujetos y las condiciones institucionales presentes en los servicios de salud.

De este modo, los espacios colectivos identificados por los residentes favorecen aprendizajes construidos a partir de la experiencia compartida del trabajo en salud, lo que permite que la colaboración interprofesional surja de la interacción continua entre diferentes sujetos, conocimientos y prácticas profesionales.

Los programas de residencia representan espacios privilegiados para experiencias colaborativas entre las diferentes profesiones de la salud, como en las atenciones compartidas, las reuniones de equipo, la tutoría y las actividades en red. Como eje formativo, la EIP potencia el desarrollo de competencias tanto individuales como colectivas. Sin embargo, su implementación efectiva aún enfrenta brechas en la comprensión conceptual, lo que exige apoyo institucional continuo, infraestructura adecuada, dominio de metodologías activas y cambios estructurales y culturales en el campo de la educación en salud, con el fin de promover la interacción entre los estudiantes de diferentes carreras, con el objetivo de romper con el paradigma del modelo curricular, hegemónicamente uniprofesional y disciplinario^(21,22)^.

La EIP depende de una intencionalidad didáctica declarada, de estrategias estructuradas y de la capacitación de los tutores y preceptores para que pueda implementarse de manera efectiva^(13)^; por ello, es fundamental contar con procesos frecuentes de educación permanente alineados con las directrices y bases de los programas que contribuyan a la formación en salud. A pesar de las oportunidades identificadas, los participantes destacaron la ausencia de una planificación pedagógica explícita orientada a la interprofesionalidad debido a la falta de preparación de los participantes (preceptores, tutores, supervisores y docentes), lo que debilita las acciones interprofesionales.

De este modo, la planificación de las actividades de enseñanza y la evaluación del proceso de enseñanza-aprendizaje son aspectos cruciales y relevantes para la adopción de la EIP. Para ello, es necesario que los proyectos pedagógicos incluyan una carga horaria específica para la realización de reuniones planificadas entre tutores, preceptores y residentes, ya que estas garantizan la intencionalidad de las actividades y reconocen la importancia de la evaluación sistemática y continua del aprendizaje y de la adquisición de competencias colaborativas, que abarcan la capacidad del estudiante para actuar en conjunto con otros profesionales, usuarios, familias y comunidades^(23)^.

En este sentido, la escasa interacción entre tutores, preceptores y residentes también puede constituir un obstáculo para la comprensión de la interprofesionalidad por parte de los residentes, ya que las reuniones entre ellos podrían orientarse según la EIP y la PCI, aspectos que en los programas analizados se exploran de manera insuficiente. Así, las experiencias colaborativas desarrolladas en la residencia se muestran directamente relacionadas con la calidad de las interacciones que se producen en el día a día del trabajo en salud y con las posibilidades de construir de manera compartida la atención entre los diferentes sujetos involucrados.

El aprendizaje en grupos pequeños con una representación equilibrada de todas las categorías profesionales involucradas se presenta como otra estrategia para facilitar el aprendizaje interprofesional^(23)^, algo que solo se observó en el PRMSM, ya que contaba con un número equitativo de cupos por categoría profesional. Este hallazgo señala que la configuración de los entornos de práctica tiene un impacto directo en la materialización de la interprofesionalidad.

Las prácticas interprofesionales permitieron a los residentes reconocer los conocimientos de sus colegas, reflexionar críticamente sobre la atención y fortalecer el trabajo en equipo, promoviendo una formación integrada y centrada en las necesidades del usuario. Dichos aprendizajes van más allá de la dimensión técnico-operativa, ya que involucran procesos relacionales y comunicativos construidos a partir de las experiencias compartidas de la atención y el trabajo en salud. De este modo, la EIP y la PCI se configuran como enfoques prometedores para mejorar la atención integral al desarrollar competencias comunes, específicas y colaborativas, complementando —y no sustituyendo— los modelos multiprofesionales, multidisciplinarios, interdisciplinarios o incluso uniprofesionales^(24)^.

Los PRMS representan una oportunidad para romper los estereotipos profesionales, como se evidenció en los encuentros, destacando la importancia de la claridad de los roles para un trabajo colaborativo eficaz y la reconstrucción de los conocimientos específicos de cada profesión. El reconocimiento de la interdependencia entre las áreas refuerza la importancia de la comunicación interprofesional como competencia esencial para superar barreras, fortalecer relaciones y mejorar la atención mediante decisiones compartidas^(25)^.

El desconocimiento sobre el papel de algunas profesiones se hizo evidente en el discurso de los participantes. Algunas profesiones, al ser históricamente más antiguas, son mejor comprendidas, aunque de manera estereotipada por los diversos sectores de la sociedad, como la enfermería y la medicina. Otras, como la terapia ocupacional, por ejemplo, generan extrañeza entre las demás profesiones y los usuarios respecto a su función profesional^(26)^.

Algunas categorías profesionales, que forman parte de los programas aquí analizados, aparecen en la literatura como las que presentan mayores dificultades para integrarse en los espacios de los servicios, como la fonoaudiología, la nutrición, la psicología y la terapia ocupacional. Esta situación se ve agravada por las expectativas depositadas en estos profesionales, principalmente en aquellos que prestan servicios especializados de manera individualizada, lo cual contrasta con la lógica interprofesional y requiere una mayor comprensión de los roles y una alineación de los objetivos para lograr una integración efectiva de estas categorías en las prácticas de salud^(27)^.

Existen diferentes percepciones respecto a la existencia de áreas profesionales más abiertas y receptivas al EIP. Hay instrumentos, como la Escala de Preparación para el Aprendizaje Interprofesional (*Readiness for interprofessional Learning Scale* – RIPLS), que miden la preparación de los estudiantes para el aprendizaje compartido con diferentes categorías profesionales, evaluando tres factores: el trabajo en equipo y la colaboración, la identidad profesional y la atención de salud centrada en el paciente^(28)^. Sin embargo, existen divergencias en la literatura entre las escalas utilizadas en los estudios, lo que dificulta la comparación entre los hallazgos que analizan a los estudiantes y profesionales de manera longitudinal durante y después de la realización de una intervención^(23,29)^.

En una investigación que tuvo como objetivo conocer las experiencias vividas por los egresados de un PRMSF, se identificó que el programa de residencia se presenta como un escenario para aprender del otro y comprender el papel del otro^(14,30)^. Por su parte, un estudio que analizó la integración de los programas de residencia médica (PRMS) en la configuración de las redes de atención de salud (RAS) constató que estos programas favorecen la puesta en práctica del trabajo en red, lo que pone de manifiesto su importancia para la formación profesional^(30)^.

La presencia de los residentes en el día a día de los entornos de práctica da lugar a un aumento de la fuerza laboral de los servicios y de la capacidad del equipo para resolver los problemas de salud de los usuarios; al mismo tiempo, genera confusión en cuanto a cuáles serían sus roles en los entornos de formación, ya que se les considera una posibilidad para suplir la falta de personal de salud, lo que se desvía de la premisa de los PRMS^(30)^.

A esta cuestión se suman otros factores que inciden en la enseñanza y el aprendizaje de los PRMS, entre los que se incluyen la divergencia entre la propuesta pedagógica y su aplicación práctica, la desconexión entre el plan de estudios y las actividades de campo, los conflictos interpersonales, los estereotipos, la falta de reconocimiento de los roles, la resistencia a la EIP, los modelos tradicionales de formación, la escasa capacitación de los tutores, la supervisión limitada, la comunicación ineficaz y la valorización del desempeño individual y la competitividad^(11,30)^. Estos factores demuestran que la consolidación de la interprofesionalidad depende no solo de la disposición individual de los profesionales y residentes, sino también de las condiciones organizativas, pedagógicas e institucionales que estructuran los procesos de trabajo y formación en salud. Para cambiar esta realidad, se requiere un esfuerzo conjunto de las instituciones de formación, los servicios de salud y las políticas públicas de salud y educación que valoren la EIP como base de la formación en salud.

Las limitaciones de esta investigación se derivan de que la investigación se llevó a cabo en una sola institución que ofrece programas de residencia médica (PRMS), así como de la falta de participación de preceptores, tutores, coordinadores, usuarios y profesionales de los servicios, lo que impide el análisis del contexto a partir de la experiencia de todos los sujetos involucrados en los escenarios de práctica y formación de los programas de residencia. A esto se suma el hecho de que la recolección de datos se realizó mediante reuniones remotas y sincrónicas, lo que pudo haber influido en la interacción entre los investigadores y los participantes, así como en la captación de elementos no verbales de la comunicación.

Los hallazgos de este estudio ponen de manifiesto avances relevantes para la enfermería y el ámbito de la salud al reafirmar los PRMS como escenarios estratégicos para el desarrollo de la educación y la práctica colaborativa interprofesional. Al evidenciar aprendizajes relacionados con la comunicación interprofesional, el reconocimiento de roles y la toma de decisiones compartida, el estudio contribuye al fortalecimiento de competencias esenciales para la atención integral en el SUS. Para el ámbito de la salud, los hallazgos amplían la comprensión sobre los factores pedagógicos, organizativos e institucionales que favorecen o limitan la interprofesionalidad en los entornos de formación en el servicio, ofreciendo aportaciones para el perfeccionamiento de proyectos pedagógicos, políticas de educación continua y modelos de gestión del trabajo en salud.

Además, los estudios que analizan los procesos formativos y las dinámicas interprofesionales en los programas de residencia tienen un carácter estructurante y no meramente circunstancial, ya que abordan dimensiones organizativas, pedagógicas y relacionales que tienden a mantenerse a lo largo del tiempo, incluso ante cambios coyunturales. Por lo tanto, los resultados obtenidos en esta investigación siguen siendo actuales y relevantes, ya que contribuyen al avance del conocimiento en el área, respaldan la mejora de los programas de residencia y apoyan la formulación y el perfeccionamiento de políticas públicas orientadas a la formación en salud.

## CONSIDERACIONES FINALES

La presente investigación permitió conocer las experiencias de educación y práctica colaborativa interprofesional de los residentes en programas de residencia multiprofesional en salud, poniendo de manifiesto las oportunidades formativas, los aprendizajes desarrollados, los componentes necesarios y los desafíos para lograr la interprofesionalidad en los entornos de formación en servicio. Los hallazgos mostraron que estas experiencias se materializan principalmente en espacios cotidianos de interacción entre los residentes y los equipos de los servicios, como atenciones compartidas, discusiones de casos y reuniones de equipo, lo que favorece el reconocimiento de los roles profesionales y el desarrollo de competencias colaborativas.

Sin embargo, los resultados también pusieron de manifiesto que la consolidación de la interprofesionalidad sigue viéndose afectada por aspectos estructurales, organizativos y pedagógicos de los servicios y programas de residencia, como el predominio de la lógica multiprofesional en la formación y el trabajo en salud, la elevada demanda de atención en los servicios y la falta de una planificación intencional de las actividades interprofesionales. Por lo tanto, es imperativo que la EIP se implemente en los diferentes procesos formativos, rompiendo con los paradigmas tradicionales de la enseñanza y el trabajo en salud, con el fin de mejorar la atención de salud y la colaboración entre los profesionales.

Por último, se destaca la importancia de las investigaciones que consideren diferentes contextos, enfoques y poblaciones, especialmente los estudios longitudinales, de intervención y de métodos mixtos, que aún son escasos en el ámbito de la educación y la práctica interprofesional. Se cree que nuevas investigaciones podrán contribuir a cambios en la formación y en la formulación de nuevas políticas públicas de salud y educación.

## Data Availability

Todo el conjunto de datos que respalda los resultados de este estudio se publicó en el propio artículo.
